# Long‐Term Changes in the Winter Diet of Common Dolphins Reflects Ecological Shifts and Bycatch Dynamics in the Bay of Biscay

**DOI:** 10.1002/ece3.71815

**Published:** 2025-07-18

**Authors:** Johanna Faure, Jasmin Niol, Eléonore Meheust, Jérôme Spitz

**Affiliations:** ^1^ Observatoire Pelagis UAR 3462 CNRS – La Rochelle Université La Rochelle France; ^2^ Centre D'etudes Biologiques de Chizé (CEBC) UMR 7372 CNRS – La Rochelle Université Villiers‐en‐Bois France

**Keywords:** marine mammals, small schooling fish, stomach contents, temporal variation, trophic ecology

## Abstract

Bycatch (e.g., the accidental capture of non‐targeted species by fisheries) is a leading cause of human‐induced mortality, contributing to significant population declines worldwide. Often stemming from the overlap between food resources and fishery target species, dietary analysis is key to understanding bycatch patterns. In the Bay of Biscay, common dolphin ( *Delphinus delphis* ) bycatch has strongly increased since 2016. Addressing the potential trophic relationship between dolphins and fisheries is essential for developing effective conservation strategies to ensure the sustainability of both dolphin populations and fisheries. Using stomach content analysis, we investigated temporal changes in the occurrence, abundance, and importance by mass of prey between 1999 and 2019. We found no difference in overall diet over time, still composed of pelagic energy‐rich prey (pilchards *Sardina pilchardus* ; horse mackerel *Trachurus* spp. and anchovy *Engraulis encrasicolus* ). However, we observed a significant decrease in the importance by mass of horse mackerel (*t* = 2.8365, *p* < 0.01) and an increase in anchovy (*t* = −4.2636, *p* < 0.01), as well as a decrease in the average size of major species, mainly related to environmental variations in abundance and size distribution of the small pelagic fish. We also identified a shift in minor species from upper slope habitats (e.g., blue whiting *Micromesistius poutassou* ) to species inhabiting coastal waters (e.g., sprat *Sprattus sprattus* ), reflecting changes in the distribution of common dolphins within the Bay of Biscay. Finally, we highlighted the consistency over time in the prevalence of fresh pilchards and anchovies in the dolphin stomachs, suggesting they are more likely to feed specifically on these species when bycatch occurs. The risk of bycatch may therefore be increased by dolphins targeting pilchards and anchovies in more coastal waters.

## Graphical abstract

First study investigating temporal changes in the diet of the common dolphin ( *Delphinus delphis* ) in the Bay of Biscay between 1999 and 2019, we found that the diet of the common dolphin remained mainly composed of small energy‐rich fish species. However, we observed a shift in minor prey from upper slopes habitats to coastal waters reflecting a shift of the feeding zone nearer to the coast associated with a decrease in the average size of major preys.

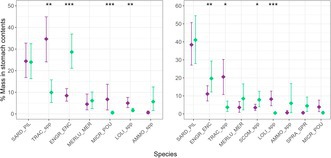

## 1 Introduction

Interactions between fisheries and marine megafauna, such as sharks, seabirds, and marine mammals, have long existed in all oceans (Crowder et al. 2008; Furness 2003; Lewison et al. 2004; Northridge 1991). These interactions can be indirect—through fishing‐induced changes in prey abundance and size structure (Demaster et al. 2001; Goñi 1998; Heino et al. 2015; Hsieh et al. 2006; Jennings and Kaiser 1998)—or direct, through incidents like ship strikes, entanglements, and captures in fishing gear (Lewison et al. 2004; Read 2008; Schoeman et al. 2020; Stelfox et al. 2016). Notably, the accidental capture of non‐targeted species by fisheries, known as bycatch, is a leading cause of human‐induced mortality in marine megafauna, contributing to significant population and species declines worldwide (Avila et al. 2018; Barbraud et al. 2013; Dulvy et al. 2014; Lewison et al. 2004; Read 2008; Žydelis et al. 2013). These large marine vertebrates are particularly susceptible to fishing‐related mortality due to their life‐history traits, including long lifespans, low reproductive rates, and late maturity (Davidson et al. 2012; Dulvy et al. 2014; Hutchings et al. 2012). Thus, managing the environmental impacts of fishing, including bycatch, has become crucial (Gilman et al. 2022; Hazen et al. 2018; Lewison et al. 2014; Soykan et al. 2008).

Among marine mammals, bycatch frequently occurs in both commercial and artisanal fisheries (Jog et al. 2022). This problem often arises from the overlap between the species targeted both by these marine predators and by fisheries, leading to direct competition for resources, or at least spatial overlap between predator foraging distribution and fishing areas (Baird et al. 2021; Ferro de Godoy et al. 2020; Morissette et al. 2012). Consequently, bycatch rates can be influenced by the foraging strategies of the different predator species, and the commercial species targeted by fisheries, as well as the fishing gears used. The understanding of feeding habits and trophic interactions of predators is therefore essential to shed light on their interactions with fisheries (Young et al. 2015). Stomach content analysis is commonly used in feeding ecology studies of marine predators (Bowen and Iverson 2013; Trites and Spitz 2018). Despite potential biases due to variable digestion rates and rare prey, this method provides critical details on the prey species consumed, including individual prey size and mass (Amundsen and Sánchez‐Hernández 2019). Unlike other techniques, such as stable isotope analysis, which offer a more integrated view (Newsome et al. 2010; Trites and Spitz 2018), stomach content analysis can focus on the most recent food intake by using only fresh remains, thus making it particularly relevant to understand some mechanisms leading to bycatch events.

The Bay of Biscay in the northeastern Atlantic Ocean is a highly productive region, characterized by upwelling areas that support a rich abundance and diversity of fish species (Allain et al. 2001; Borja et al. 2019). Hence, numerous fisheries have intensively operated over time using a variety of fishing gear such as gillnets, trawls, and trammel nets (Demanèche et al. 2019; Guénette and Gascuel 2012). This productive ecosystem also sustains numerous top predators, including marine mammals, of which the short‐beaked common dolphin ( *Delphinus delphis* ) is the most abundant (Gilles et al. 2023; Laran et al. 2017; Perrin 2018). To meet their high‐energy requirements, common dolphins feed primarily on small energy‐rich pelagic schooling fish, such as anchovy ( *Engraulis encrasicolus* ), pilchards ( *Sardina pilchardus* ) or horse mackerel (*Trachurus* spp.), which are also important fisheries targets (Meynier et al. 2008; Spitz et al. 2014; Spitz et al. 2018). However, common dolphins have been regularly caught as bycatch for decades, initially mostly in winter in the pelagic trawl fishery targeting seabass ( *Dicentrarchus labrax* ) (Castro et al. 2024; Morizur et al. 1999; Peltier et al. 2016). Since 2016, a strong increase in the bycatch level of common dolphins has been observed in the Bay of Biscay (Meheust et al. 2021; Peltier et al. 2021). In addition, these high levels of bycatch have been found to occur not only in pelagic trawls but also in gillnets and trammel nets targeting flatfish and European hake, *Merluccius merluccius* (Paillé et al. 2024).

This occurred in conjunction with a change in the spatial distribution of the common dolphin, more widespread and nearer to the coast than before (Laran et al. 2022). However, the detailed mechanisms underlying these changes remain to be identified.

Common dolphins' bycatch is recognized as a major issue for fisheries management and conservation in Europe. The level of this additional mortality is estimated as unsustainable for the long‐term viability of this cetacean (Taylor et al. 2022) and in the Bay of Biscay, a one‐month closure of the fisheries concerned has been decreed during the winter period. Understanding the mechanisms leading to their capture is essential to implementing long‐term effective mitigation measures allowing both to maintain the dolphin populations and fisheries. Stomachs of bycaught individuals generally contain fresh prey remains, providing evidence that bycatch occurs when feeding and is therefore related to trophic interactions. Trophic overlap in the feeding preferences of sea bass and common dolphins has previously been identified as an underlying mechanism to explain the high bycatch rates observed in pelagic trawl fisheries in the Bay of Biscay when common dolphins feed among sea bass schools (Spitz et al. 2013); however, any data on common dolphin diet is available since 15 years (Meynier et al. 2008). The recent increase in dolphin bycatch, notably in bottom nets, associated with changes in their spatial distributions, could be related to modifications in their feeding ecology. Therefore, using data from stomach content analysis conducted between 1999 and 2019, we investigated temporal changes in the feeding ecology of the common dolphin in the Bay of Biscay. The main objectives of this study were to (i) explore differences in prey composition and diversity and (ii) analyze prey‐specific differences in size and relative importance over time. Finally, we discussed the ecological implications of our results in the context of bycatch mechanisms and changes in prey species availability.

## 2 Materials and Methods

### 2.1 Data Collection

**FIGURE 1:**
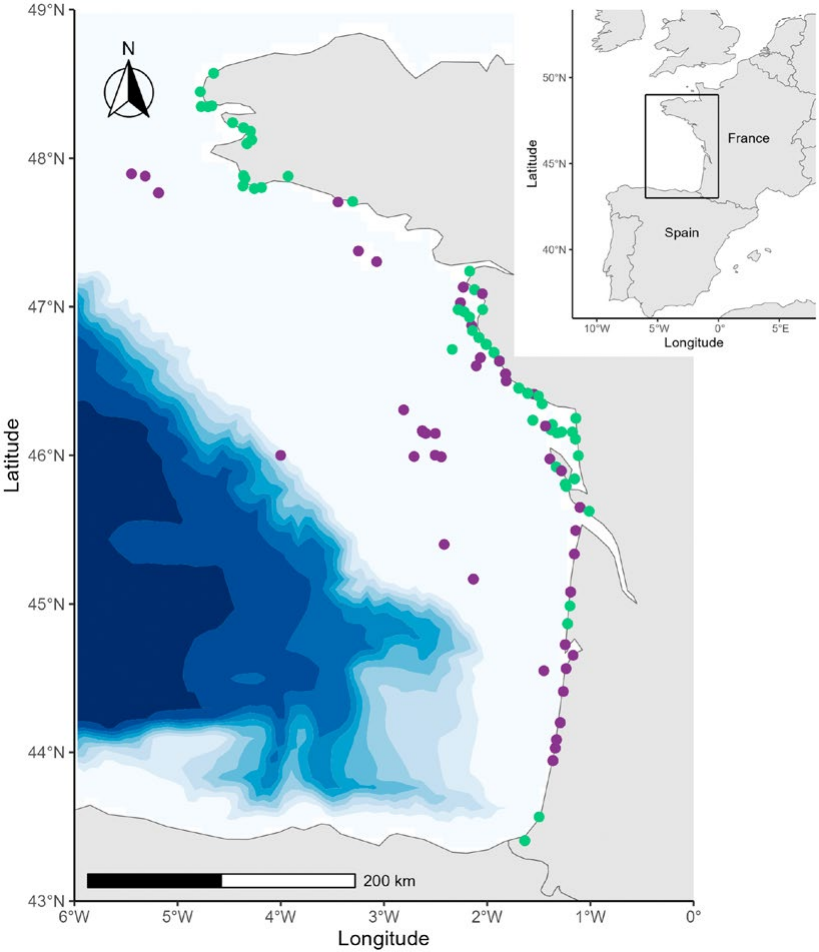
Spatial location of the study area (left panel) and sites where the samples of *Delphinus delphis* were collected (right panel) between 1999 and 2006 (purple) and 2017–2019 (green).

A total of 255 stomach contents of bycaught common dolphins were collected in the Bay of Biscay during winter (December to March) from 1999 to 2006 and from 2017 to 2019 (Figure 1). Twenty‐eight individuals were directly sampled on board pelagic pair trawlers in 2005 and 2006, having already died on arrival on deck. The remaining stomach contents were sampled from dolphins found stranded along the French coast of the Bay of Biscay, whose cause of death was attributed to a bycatch event (*n* = 227) by the French national stranding network (Wund et al. 2023). In both cases, sampling was done within the framework of the French regulations and the French Stranding Network with the necessary authorizations.

Individuals were dissected either in the field or in the laboratory, stomachs were ligatured, and stored deep‐frozen (−20°C) in polythene bags awaiting further analysis. Each individual has been measured and sexed, and the location of capture or stranding was collected (Table 1). 17 individuals were excluded from further analysis because their size or sex remained undetermined, including 3 that were found stranded with their tails cut off. Data from 68 individuals, collected between 1999 and 2002, have previously been included in a scientific publication (Table 1; Meynier et al. 2008). All individuals were adults, and a total of 126 females and 112 males were sampled.

**TABLE 1:** Number of samples, percentage of females, mean body size (±SD) and ranges (cm) of individuals per year and per time period.

Year	Nb samples	% Females	Body size	Range	Source
1999	13	38.5	188 ± 30	[128–233]	Meynier et al. (2008)
2000	19	52.6	187 ± 18.2	[158–232]	Meynier et al. (2008)
2001	15	33.3	183 ± 29.0	[132–228]	Meynier et al. (2008)
2002	21	57.1	194 ± 20	[147–223]	Meynier et al. (2008)
2003	12	58.3	183 ± 17.9	[144–202]	This study
2004	12	66.7	199 ± 20.8	[174–243]	This study
2005	9	66.7	181 ± 21.9	[150–221]	This study
2006	21	72.00	181 ± 20.9	[138–225]	This study
**Total**	122	56.5	187 ± 22.6	[128–243]	
2017	48	52.1	184 ± 22.5	[110–224]	This study
2018	38	50.0	183 ± 22.9	[109–228]	This study
2019	30	43.3	183 ± 19.0	[120–222]	This study
**Total**	116	49.1	183 ± 21.6	[109–228]	

### 2.2 Stomach Content Analysis

Whole stomachs were thawed and their content was washed through a sieve of 0.2 mm mesh size. From the 238 samples analyzed, hard diagnostic parts (fish bones, otoliths, and cephalopod beaks) and other prey items were retrieved and identified by using published guides (e.g., Clarke 1986; Härkönen 1986) or an internal reference collection. Fish bones and otoliths were stored dry, whereas cephalopod beaks and crustacean remains were kept in 70% ethanol. Each prey item was sorted as accumulated (i.e., hard parts without flesh attached) or as fresh (i.e., when flesh remained attached to hard parts), allowing us to determine a fresh fraction. The fresh fraction is deemed to be more representative of the composition of prey consumed close to death than a total diet composition derived from all prey items. The total number of food items was estimated as the highest number, given by either the number of paired structures (e.g., otoliths, opercula, and hyomandibular, dentary and premaxillary bones for fishes, upper and lower beaks for cephalopods, and eyes for crustaceans) or unpaired structures (e.g., parasphenoid bone for fishes, gladius for cephalopods, and carapace and telson for crustaceans). Diagnostic hard parts were measured (±0.02 mm) following standards. If more than 30 remains were present for one taxon in a stomach, a sub‐sample of 30 was measured. Individual prey body length (total length—TL—for fish and Dorsal Mantle Length for cephalopods, in cm) and mass (in g) were calculated using regressions from the literature or from our reference collection (Table S1).

### 2.3 Diversity and Composition Analyses

Temporal variation was investigated considering two periods: “former” including individuals sampled between 1999 and 2006 and “recent” between 2017 and 2019. Diet analyses were conducted according to two levels of detail: *Total diet*, including all data regardless of the prey items' state of digestion, and *Fresh diet*, only including fresh items. 15 stomach contents only contained accumulated prey items and were only considered in the *Total diet* analysis. Thus, for *Total diet* and *Fresh diet* analyses, a total of 238 and 223 stomach contents were statistically analyzed using R (version 4.3; R Core Team 2023).

Cumulative prey curves were constructed against the randomly pooled number of analyzed stomachs to check if a sufficient number of stomach contents had been collected to accurately describe the diet of each predator (Cortés 1997). Curves were generated after 100 randomizations of the original data using the Vegan Community Ecology package (Oksanen et al. 2024). When curves approached an asymptote, it was considered that a sufficient number of stomachs had been processed. To statistically assess the adequacy of sample size, a linear regression was performed on the final five points of the curve. The leveling off of the prey curve was considered acceptable when the slope was *b* < 0.05. To test temporal dietary differences, time periods were separated.

Frequencies of occurrence, relative abundance, and reconstructed biomass of each prey species were calculated by time period. The frequency of occurrence of a given prey taxon was calculated as the number of stomachs in which the taxon was observed. The relative abundance was assessed as the number of items found in the sample set. The reconstructed biomass was calculated as the product of the number of individuals and the average reconstituted body mass in each stomach, summed throughout the sample set. Sampling errors were assessed by generating non‐parametric 95% confidence intervals around percentage number and mass using bootstrap simulations, where random samples were drawn with replacement with 1000 iterations (matrices produced will be available on InDoRES data repository by the time of the publication).

Total fish length distributions per period were weighed by the reconstituted mass. Fish length distributions at the sample level were weighted by the number of individuals in the sample and summed to produce the overall size distribution of a prey taxon in the whole series of samples.

Within and between samples, diversity was investigated using the alpha diversity index, including estimates of species richness, Shannon's and Simpson's diversity indices based on abundance to investigate evenness and equitability of species in each sample (Peet 1975). These analyses were performed over prey occurrence and abundance data between time periods for *Total diet* and *Fresh diet* analyses.

### 2.4 Diet Comparisons

#### 2.4.1 Composition

Comparison of diet composition (reconstructed biomass of each prey species) between time periods was assessed using nonmetric multidimensional scaling (nMDS) ordination based on Bray‐Curtis dissimilarities on log‐transformed biomass data. Ordination mean plots were constructed through bootstrap average (*n* = 100). We also tested the correlation of spatial and biological variables (longitude and latitude of sampling and size and sex of dolphins) with the ordination configuration to investigate drivers for the composition of dietary samples and test for sensibility to sampling. The significance of fitting vectors was assessed using a permutation of environmental variables (*n* = 999). Analyses of similarities (ANOSIM) also based on Bray‐Curtis dissimilarities, were used to test the significance of the observed patterns in the nMDS (Somerfield et al. 2021). nMDS and ANOSIM were performed using the Vegan Community Ecology package (Oksanen et al. 2024).

To assess the degree of diet overlap between the two periods, we used Pianka's Index using the function *dietOverlap()* in the R package ‘FSAmisc’ (Ogle et al. 2025).

At the species level, to determine whether there was a significant difference in the contribution to mass of each prey among time periods, we performed a Student t‐test (*t* statistic) over diet row data. When the normality of data was not validated, the non‐parametric test Wilcoxon (*W* statistics) was used.

#### 2.4.2 Prey Size

After normality was validated, comparison of fish length distributions was performed using Student's *t*‐test weighted by the number of individuals with the Survey package (Lumley 2004).

## 3 Results

Cumulative prey curves indicated sample size reach asymptote (*b* < 0.05) at the species level for both time periods (Figure S2). Sample sizes were thus sufficient to describe the overall and fresh diet of the common dolphin.

### 3.1 Descriptive Analysis and Composition of Diet

#### 3.1.1 Former Period (1999–2006)

To describe the diet of the common dolphin in the *former* period, a total of 122 individuals were sampled from 1999 to 2006 on the French coast of the Bay of Biscay (Figure 1). These individuals included 67 females and 55 males and ranged from 128 to 243 cm (Table 1). Out of these 122 stomach contents, 28,966 prey items were retrieved, including the presence, on average, of 237 (±336 SD) prey items in each stomach. Among all prey items, 21,348 were considered accumulated and were not included in fresh diet analysis (i.e., 6 stomach contents, only containing accumulated items, were excluded). Overall, 95% of the stomachs showed the presence of fresh remains.

Fish were predominant in both total and fresh diet analyses, respectively occurring in 100% and 92% of samples analyzed (Table 2). Fish were also prevalent by number and reconstructed mass in total diet (92.6% and 92.8%, respectively) and in fresh diet (78.2% and 86.4%, respectively). In comparison, cephalopods occurred in 94 of the samples (77%) (including 70% of the samples containing only fresh items) and ranked second by number in total (5.6%) and fresh diet (16.4%) but also by reconstructed mass (7.2% and 13.6%, respectively). Crustaceans were only anecdotal in both total and fresh diets.

**TABLE 2:** Frequency of occurrence (%FO), relative abundance (%N) and reconstructed biomass (%M) of prey items identified from stomach contents of common dolphin pooled in the former period (1999–2006) for the total diet (*N* = 122) and the fresh diet (*N* = 116).

Prey species	Total diet					Fresh diet				
	Occurrence	Number		Reconstructed biomass		Occurrence	Number		Reconstructed biomass	
	%FO	%N	95% CI	%M	95% CI	%FO	%N	95% CI	%M	95% CI
**Fish**	**100**	**92.6**		**92.8**		**92.2**	**78.2**	—	**86.4**	—
Argentinidae										
*Argentina* spp.	10.6	0.4	[0.1–0.7]	0.5	[0.2–0.9]	3.4	0.2	[0.01–0.5]	0.3	[0.02–0.7]
Bathylagidae										
Unidentified Bathylagidae	0.8	0.06	[0–0.2]	0.6	[0–2.0]	—	—	—	—	—
Atherinidae										
*Atherina presbyter*	3.2	0.1	[< 0.01–0.2]	0.07	[< 0.01–0.2]	—	—	—	—	—
Belonidae										
*Belone belone*	1.6	0.01	[0–0.04]	0.3	[0–0.9]	1.7	0.05	[0–0.2]	1.0	[0–2.8]
Carangidae										
*Trachurus* spp.	80.3	24.8	[15.5–35.0]	34.7	[23.1–45.5]	40.5	21.2	[11.0–32.8]	20.7	[10.8–31.0]
Alosidae										
*Sardina pilchardus*	62.3	4.8	[3.0–7.1]	24.2	[16.7–32.3]	44.8	10.3	[6.0–16.2]	38.4	[26.4–50.0]
Clupleidae										
*Sprattus sprattus*	8.2	0.3	[0.05–0.5]	0.3	[0.05–0.6]	4.3	0.6	[0.1–1.2]	0.6	[0.08–1.3]
Engraulidae										
*Engraulis encrasicolus*	69.7	12.7	[8.8–17.6]	8.5	[5.9–11.8]	54.3	19.7	[13.0–27.0]	11.1	[7.4–15.5]
Unidentified Clupeiformes	0.8	0.02	[0–0.06]	0.1	[0–0.5]	—	—	—	—	—
Labridae										
Unidentified Labridae	1.6	0.01	[0–0.03]	0.01	[0–0.04]	0.9	0.01	[0–0.05]	< 0.01	[0–0.03]
Sparidae										
Sparidae spp.	3.2	0.02	[< 0.01–0.05]	0.05	[< 0.01–0.1]	2.6	0.06	[0–0.2]	0.1	[0–0.3]
Gadidae										
*Gadiculus argenteus*	3.2	0.02	[< 0.01–0.06]	0.01	[< 0.01–0.03]	—	—	—	—	—
*Merlangius merlangus*	4.0	0.2	[0.01–0.5]	1.3	[0.08–3.0]	—	—	—	—	—
*Micromesistius poutassou*	36.9	3.6	[1.0–7.5]	6.8	[1.8–14.0]	13.8	1.5	[0.5–3.0]	3.6	[1.1–7.6]
*Trisopterus* spp.	48.4	2.6	[1.1–4.3]	4.0	[1.6–7.5]	13.8	1.5	[0.6–2.8]	1.8	[0.6–3.7]
Unidentified Gadidae	15.6	0.2	[0.08–0.4]	0.05	[0.02–0.09]	—	—	—	—	—
Lotidae										
*Molva molva*	0.8	< 0.01	[0–0.02]	0.2	[0–0.7]	—	—	—	—	—
Gaidropsaridae (Rockling)										
Unidentified Gaidropsaridae	9.0	0.03	[0.08–0.5]	0.2	[0.07–0.4]	—	—	—	—	—
Merlucciidae										
*Merluccius merluccius*	32.8	1.2	[0.5–2.4]	4.6	[1.7–9.2]	11.2	1.0	[0.2–1.9]	3.7	[0.8–7.1]
Gobiidae										
Unidentified Gobiidae	84.4	40.2	[30.4–45.2]	3.2	[2.0–4.6]	19.0	19.6	[9.3–30.8]	1.0	[0.4–1.7]
Mugilidae						—	—	—	—	—
Unidentified Mugilidae	0.8	< 0.01	[0–0.01]	0.02	[0–0.06]					
Myctophidae						—	—	—	—	—
*Benthosema glaciale*	0.8	< 0.01	[0–0.01]	< 0.01	[0 to < 0.01]					
*Myctophum punctatum*	2.4	0.07	[0–0.2]	0.01	[0–0.04]	0.9	0.04	[0–0.1]	< 0.01	[0–0.02]
*Notoscopelus kroeyeri*	1.6	0.03	[0–0.07]	< 0.01	[0–0.02]	—	—	—	—	—
Ammodytidae										
Unidentified Ammodytidae	9.0	0.4	[0.09–0.8]	0.6	[0.1–1.4]	4.3	0.6	[0.03–1.6]	0.7	[0.03–2.0]
**Flatfish**										
Pleuronectidae										
Unidentified Pleuronectidae	4.0	0.02	[< 0.01–0.05]	0.04	[< 0.01–0.09]	0.9	0.01	[0–0.04]	0.02	[0–0.06]
Soleidae										
*Dicologlossa cuneata*	0.8	< 0.01	[0–0.02]	< 0.01	[0–0.02]	—	—	—	—	—
*Microchirus* sp. /*Buglossidium* sp.	0.8	< 0.01	[0–0.02]	0.02	[0–0.06]	—	—	—	—	—
Unidentified flatfish	3.2	0.04	[< 0.01–0.08]	—	—	1.7	0.07	[0–0.2]	—	—
Scombridae										
*Scomber* spp.	21.3	0.3	[0.1–0.7]	2.3	[0.1–4.6]	18.1	0.6	[0.3–0.9]	3.4	[1.9–5.3]
Stomiidae										
Unidentified Stomiidae	0.8	< 0.01	[0–0.01]	—	—	0.9	0.01	[0–0.04]	—	—
Unidentified fish	5.7	0.4	[0.01–1.1]	—	—	0.9	1.1	[0–4.0]	—	—
**Cephalopods**	**77.0**	5.6	—	**7.2**	—	**69.8**	**16.4**	—	**13.6**	—
Squids										
Brachioteuthidae										
*Brachioteuthis* spp.	0.8	< 0.01	[0–0.01]	< 0.01	[0 to < 0.01]	—	—	—	—	—
Cranchiidae										
*Teuthowenia megalops*	0.8	< 0.01	[0–0.03]	0.02	[0–0.07]	0.9	0.04	[0–0.1]	0.06	[0–0.2]
Histioteuthidae										
*Histioteuthis reversa*	0.8	< 0.01	[0–0.01]	< 0.01	[0 to < 0.01]	—	—	—	—	—
Loliginidae										
*Alloteuthis* spp.	35.2	0.7	[0.4–1.1]	0.5	[0.3–0.8]	24.1	2.0	[0.8–3.7]	1.0	[0.4–2.2]
*Loligo* spp.	43.4	0.9	[0.6–1.5]	5.0	[2.9–7.8]	36.2	2.7	[1.4–4.7]	8.2	[4.7–13.0]
Unidentified Loliginidae	3.2	0.02	[< 0.01–0.05]	—	—	3.4	0.08	[0.01–0.2]	—	—
Ommastrephidae						—	—	—	—	—
*Illex coindetii*	0.8	< 0.01	[0–0.01]	0.04	[0–0.1]					
*Todarodes sagittatus*	0.8	< 0.01	[0–0.02]	0.5	[0–1.8]	0.9	0.03	[0–0.1]	1.6	[0–5.6]
Unidentified Ommastrephidae	9.0	0.08	[0.04–0.2]	0.7	[0.3–1.3]	2.6	0.05	[0–0.1]	1.5	[0–3.3]
Sepiidae										
*Sepia* spp.	5.7	0.1	[< 0.01–0.4]	0.07	[< 0.01–0.2]	3.4	0.4	[0.01–1.4]	0.2	[< 0.01–0.5]
Sepiolidae										
Unidentified Sepiolidae	55.7	3.7	[1.6–6.7]	0.2	[0.07–0.4]	44.8	11.0	[3.2–25.5]	0.8	[0.2–1.8]
Octopus										
Octopodidae										
Octopodidae spp.	2.4	0.01	[< 0.01–0.02]	0.1	[0–0.2]	1.7	0.03	[0–0.07]	0.2	[0–0.4]
Unidentified cephalopods	3.2	0.02	[< 0.01–0.05]	—	—	0.9	0.03	[0–0.09]	—	—
**Crustaceans**	**3.3**	**1.5**	—	< **0.01**	—	**1.7**	**5.4**	—	**0.02**	—
Amphipoda										
Hyperiidae	0.8	1.3	[0–5.0]	< 0.01	[0–0.02]	0.9	5.3	[0–17.7]	0.02	[0–0.06]
Shrimps										
Decapoda										
*Crangon crangon*	0.8	< 0.01	[0–0.02]	—	—	0.9	0.03	[0–0.09]	—	—
Unidentified shrimp	0.8	< 0.01	[0–0.01]	—	—	—	—	—	—	—
Unidentified crustaceans	0.8	< 0.01	[0–0.01]	—	—	—	—	—	—	—
**Total**		100		100			100		100	

Among all prey items, 44 prey species were identified in the total diet. Out of them, Gobiidae and horse mackerel were the most occurrent and abundant preys, being present in 84% and 80% of the stomach contents analyzed and reaching 40.2% (95% CI: 34.4%–45.2%) and 24.8% (95% CI: 15.5%–35.0%) of preys by number, respectively. However, because of their small size, the importance of Gobiidae was only anecdotal in reconstructed mass (3.2%; 95% CI: 2.0%–4.6%). On the contrary, horse mackerel were also the most important preys in terms of reconstructed mass, reaching 34.7% (95% CI: 23.1%–45.5%) of relative importance. Pilchards and anchovy were also important preys in terms of occurrence (62% and 70% of samples, respectively) as well as relative importance by reconstructed mass (24.2% (95% CI: 16.7%–32.3%) and 8.5% (95% CI: 5.9%–11.8%)). Other common prey species were cods and hake, including the blue whiting ( *Micromesistius poutassou* ; 37% of occurrence), the pout (*Trisopterus* spp.; 48% of occurrence) and the European hake (33%), reaching respectively 6.8% (95% CI: 1.8%–14.0%), 4.0% (95% CI: 1.6%–7.5%) and 4.6% (95% CI: 1.7%–9.2%) of reconstructed mass. Among cephalopods, *Loligo* spp. and Sepiolidae were frequent preys, being present in up to 43.4% and 55.7% of stomach contents, respectively, but were less important species in terms of relative reconstructed biomass (reaching 5.0% (95% CI: 2.9%–7.8%) for *Loligo* spp.).

Only 28 prey species were still present in the fresh diet. Among them, anchovy and Sepiolidae were the most frequent preys (respectively occurring in 54% and 45% of stomach contents) but were of lesser importance in terms of relative abundance (respectively 19.7% (95% CI: 13.0%–27.0%) and 11.0% (95% CI: 3.2%–25.5%)) and reconstructed mass (respectively 11.1% (95% CI: 7.4%–15.5%) and 0.8% (95% CI: 0.2%–1.8%)). Few other species were less common but of major importance in terms of biomass, including pilchards (45% of occurrence) and horse mackerel (40% of occurrence) reaching respectively 38.4% (95% CI: 26.4%–50.0%) and 20.7% (95% CI: 10.8%–31.0%) of the reconstructed mass. It is noteworthy that *Loligo* spp. occurred in 36% of the stomach contents and represented 8.2% (95% CI: 4.7%–13.0%) of reconstructed mass.

#### 3.1.2 Recent Period (2017–2019)

In the *recent* period, a total of 116 individuals were sampled from 2017 to 2019 on the French coast of the Bay of Biscay to describe the diet of the common dolphin (Figure 1). These individuals included 57 females and 59 males and ranged from 109 to 228 cm (Table 1). Out of these 116 stomach contents, 29,051 prey items were retrieved, including the presence, on average, of 250 ± 280 prey items in each stomach. Among all prey items, 22,134 were considered accumulated and were not included in the fresh diet analysis (i.e., 9 stomach contents, only containing accumulated items, were excluded). Overall, 92% of the stomachs showed the presence of fresh remains, indicating that the majority of the animals studied died during or shortly after feeding.

As in the *recent* period, fish prevailed in both total and fresh diet analyses, occurring in at least 99% of the samples analyzed (Table 3). Fish were also major preys by number and reconstructed mass in total diet (96.1% and 94.5%, respectively) and in fresh diet (96.9% and 97.8%, respectively). In comparison, cephalopods occurred in 102 (88%) of the samples (including 50% of the samples containing only fresh items) and ranked second by number in total (3.9%) and fresh diet (2.9%) but also by reconstructed mass (5.3% and 2.2%, respectively). Crustaceans were only anecdotal.

**TABLE 3:** Frequency of occurrence (%FO), relative abundance (%N) and reconstructed biomass (%M) of prey items identified from stomach contents of common dolphin pooled in the recent period (2017–2019) for the total diet (*N* = 116) and the fresh diet (*N* = 107).

Prey species	Total diet					Fresh diet				
	Occurrence	Number		Reconstructed biomass		Occurrence	Number		Reconstructed biomass	
	%FO	%N	95% CI	%M	95% CI	%FO	%N	95% CI	%M	95% CI
**Fish**	**99.1**	**96.1**	—	**94.5**	—	**100**	**96.9**	—	**97.8**	—
Caproidae										
*Capros aper*	3.4	0.03	[< 0.01–0.07]	0.01	[< 0.01–0.03]	0.9	0.06	[0–0.2]	0.01	[0–0.03]
Congridae										
*Conger conger*	0.9	< 0.01	[0–0.03]	0.2	[0–0.8]	0.9	0.02	[0–0.05]	0.2	[0–0.6]
Argentinidae										
*Argentina* spp.	5.2	0.08	[0.02–0.2]	0.1	[0.03–0.2]	0.9	0.02	[0–0.05]	0.02	[0–0.05]
Atherinidae										
*Atherina presbyter*	9.5	1.8	[0.4–3.5]	1.3	[0.2–2.9]	3.7	0.4	[0.01–1.0]	0.1	[< 0.01–0.4]
Belonidae										
*Belone belone*	5.2	0.03	[< 0.01–0.06]	0.8	[0.2–1.5]	4.7	0.09	[0.02–0.2]	1.2	[0.2–2.3]
Callionymidae										
*Callionymus* spp.	2.6	0.01	[0–0.02]	< 0.01	[0 to < 0.01]	1.9	0.03	[0–0.08]	< 0.01	[0 to < 0.01]
Carangidae										
*Trachurus* spp.	52.6	6.4	[3.5–10.0]	9.9	[5.2–15.7]	32.7	5.5	[2.1–10.6]	3.7	[1.3–7.2]
Alosidae										
*Sardina pilchardus*	45.7	4.3	[2.6–6.6]	23.9	[16.4–32.5]	39.3	15.4	[9.3–23.6]	41.3	[28.8–53.6]
Clupleidae										
*Clupea harengus*	0.9	0.01	[0–0.04]	0.1	[0–0.3]	0.9	0.04	[0–0.2]	0.2	[0–0.7]
*Sprattus sprattus*	34.5	3.2	[1.6–5.0]	3.6	[1.9–5.9]	30.8	6.0	[1.8–12.6]	4.4	[1.3–9.5]
Engraulidae										
*Engraulis encrasicolus*	70.7	39.1	[29.0–49.6]	28.6	[21.1–36.9]	61.7	48.0	[34.9–60.8]	19.6	[12.1–29.3]
Unidentified Clupeiformes	3.4	0.06	[< 0.01–0.1]	0.5	[0.08–1.1]	—	—	—	—	—
Labridae										
Unidentified Labridae	8.6	0.08	[0.03–0.2]	0.1	[0.04–0.2]	7.5	0.2	[0.05–0.6]	0.1	[0.03–0.3]
Sparidae										
*Boops boops*	0.9	< 0.01	[0–0.02]	0.1	[0–0.5]	—	—	—	—	—
Sparidae spp.	0.9	< 0.01	[0–0.01]	< 0.01	[0–0.03]	0.9	0.01	[0–0.05]	0.02	[0–0.08]
Gadidae										
*Gadiculus argenteus*	1.7	1.1	[0–3.3]	0.6	[0–1.9]	1.9	3.4	[0–10.3]	1.0	[0–3.2]
*Merlangius merlangus*	6.9	0.1	[0.04–0.3]	1.0	[0.3–2.0]	2.8	0.1	[0–0.3]	0.9	[0–2.1]
*Micromesistius poutassou*	4.3	0.3	[0.02–0.7]	0.6	[0.04–1.3]	4.7	0.4	[0.05–0.8]	0.6	[0.06–1.3]
*Trisopterus* spp.	25.9	1.1	[0.4–2.0]	1.8	[0.7–3.2]	12.1	1.4	[0.2–3.0]	1.2	[0.2–2.5]
*Raniceps raninus*	0.9	< 0.01	[0–0.01]	—	—	—	—	—	—	—
Unidentified Gadidae	2.6	0.02	[< 0.01–0.04]	< 0.01	[< 0.01–0.01]	0.9	0.01	[0–0.05]	—	—
Gaidropsaridae (Rockling)										
Unidentified Gaidropsaridae	6.9	0.2	[0.03–0.3]	0.1	[0.03–0.3]	6.5	0.3	[0.06–0.7]	0.1	[0.02–0.2]
Merlucciidae										
*Merluccius merluccius*	25.9	1.5	[0.6–2.6]	6.1	[2.5–10.1]	13.1	3.5	[0.9–6.7]	8.6	[2.1–17.2]
Gobiidae	93.1	30.4	[18.6–42.4]	2.5	[1.3–4.1]	43.9	3.1	[1.8–4.7]	0.1	[0.07–0.18]
*Aphia minuta*	19.0	2.4	[1.0–4.1]	0.3	[0.1–0.5]	7.5	0.4	[0.1–0.9]	0.03	[< 0.01–0.05]
*Gobius* spp.	0.9	< 0.01	[0–0.01]	< 0.01	[0 to < 0.01]	0.9	0.01	[0–0.05]	< 0.01	[0 to < 0.01]
*Lesueurigobius friesii*	5.2	0.3	[0.05–0.7]	0.03	[< 0.01–0.06]	1.9	0.3	[0–0.8]	< 0.01	[0–0.03]
*Pomatoschistus* spp.	51.7	18.6	[10.5–27.8]	1.4	[0.7–2.3]	30.8	2.1	[1.1–3.5]	0.08	[0.04–0.1]
Unidentified Gobiidae	16.4	8.9	[1.1–22.3]	0.8	[0.09–2.3]	2.8	0.2	[0–0.6]	< 0.01	[< 0.01–0.02]
Mugilidae										
Unidentified Mugilidae	0.9	0.5	[0–1.6]	2.6	[0–7.4]	0.9	0.3	[0–0.9]	0.6	[0–2.2]
Mullidae										
*Mullus surmuletus*	0.9	0.01	[0–0.04]	0.02	[0–0.08]	0.9	0.04	[0–0.1]	0.05	[0–0.2]
Myctophidae										
*Benthosema glaciale*	1.7	0.4	[0–1.3]	0.04	[0–0.12]	—	—	—	—	—
*Ceratoscopelus maderensis*	0.9	< 0.01	[0–0.01]	< 0.01	[0 to < 0.01]	—	—	—	—	—
*Myctophum punctatum*	1.7	0.4	[0–1.2]	0.08	[0–0.3]	—	—	—	—	—
*Notoscopelus kroeyeri*	1.7	0.04	[0–0.1]	< 0.01	[0 — < 0.02]	—	—	—	—	—
Ammodytidae										
Unidentified Ammodytidae	36.2	3.6	[0.9–8.5]	5.6	[1.6–12.4]	24.30	6.5	[0.6–18.5]	5.8	[0.6–16.1]
Flatfish										
Bothidae										
*Arnoglossus* spp.	5.2	0.06	[< 0.01–0.1]	0.03	[< 0.01–0.06]	1.9	0.2	[0–0.5]	0.03	[0–0.08]
Unidentified Bothidae	0.9	< 0.01	[0–0.02]	< 0.01	[0–0.02]	—	—	—	—	—
Scophthalmidae										
Unidentified Scophthalmidae	0.9	0.02	[0–0.08]	0.01	[0–0.03]	—	—	—	—	—
Soleidae										
*Microchirus* spp. /*Buglossidium* spp.	6.9	0.04	[0.01–0.08]	0.1	[0.04–0.2]	2.8	0.06	[0–0.1]	0.1	[0–0.3]
*Pegusa lascaris*	1.7	0.02	[0–0.05]	—	—	1.9	0.06	[0–0.2]	—	—
*Solea* spp.	7.8	0.07	[0.02–0.1]	0.3	[0.1–0.7]	1.9	0.03	[0–0.08]	0.07	[0–0.2]
Unidentified flatfish	3.4	0.03	[< 0.01–0.07]	—	—	—	—	—	—	—
Scombridae										
*Scomber* spp.	30.2	0.5	[0.3–0.8]	3.6	[2.2–5.7]	28.0	1.7	[0.8–2.9]	7.8	[4.0–12.8]
Sternoptychidae										
*Maurolicus muelleri*	3.5	0.9	[0.01–2.5]	0.03	[< 0.01–0.07]	0.9	0.01	[0–0.05]	< 0.01	[0 to < 0.01]
Unidentified fish	7.8	0.04	[0.01–0.08]	—	—	0.9	0.01	[0–0.05]	—	—
**Cephalopods**	**87.9**	**3.9**	—	**5.3**	—	**49.5**	**2.9**	—	**2.2**	—
Squids										
Gonatidae										
*Gonatus* spp.	0.9	< 0.01	[0–0.01]	0.06	[0–0.2]	—	—	—	—	—
Loliginidae										
*Alloteuthis* spp.	25.9	0.3	[0.2–0.5]	0.2	[0.1–0.3]	8.4	0.2	[0.08–0.3]	0.07	[0.03–0.1]
*Loligo* spp.	25.9	0.3	[0.2–0.4]	1.7	[1.0–2.6]	10.3	0.3	[0.1–0.4]	0.5	[0.2–0.9]
Unidentified Loliginidae	8.6	0.05	[0.02–0.09]	—	—	—	—	—	—	—
Ommastrephidae										
Unidentified Ommastrephidae	10.3	0.2	[0.04–0.5]	1.6	[0.4–3.8]	1.9	0.04	[0–0.1]	0.9	[0–2.2]
Sepiidae										
*Sepia* spp.	13.8	0.01	[0.06–0.2]	0.09	[0.04–0.1]	12.1	0.3	[0.1–0.6]	0.09	[0.04–0.2]
Sepiolidae										
Unidentified Sepiolidae	75.9	2.9	[1.8–4.2]	0.2	[0.1–0.2]	34.6	2.0	[1.2–3.0]	0.1	[0.06–0.1]
Octopus										
Octopodidae										
Octopodidae spp.	15.5	0.1	[0.06–0.2]	1.4	[0.7–2.2]	4.7	0.1	[0.03–0.2]	0.5	[0.07–0.9]
Unidentified cephalopods	—	—	—	—	—	—	—	—	—	—
**Crustaceans**	**1.7**	**0.04**	—	—	—	**1.9**	**0.2**	—	—	—
Shrimps										
Decapoda										
*Crangon crangon*	0.9	0.04	[0–0.1]	—	—	0.9	0.2	[0–0.5]	—	—
Unidentified shrimp	0.9	< 0.01	[0–0.01]	—	—	0.9	0.01	[0–0.05]	—	—
**Total**		**100**		**100**			**100**		**100**	

Among all prey items, 53 prey species were identified in the total diet. Out of them, anchovy was the most common and abundant prey, being present in 71% of the stomach contents analyzed and reaching 39.1% (95% CI: 29.0%–49.6%) and 28.6% (95% CI: 21.1%–36.9%) of relative abundance and reconstructed mass. Sepiolidae was also a common species with 76% of occurrence through the samples, but represented only a small part of relative abundance and mass (respectively 2.9% (95% CI: 1.8%–4.2%) and 0.2% (95% CI: 0.1%–0.2%)). As in the *former* period, Gobiidae, including *Pomatoschistus* spp., and horse mackerel were frequent species, being both present in 52% of the samples analyzed but were only of secondary importance in terms of relative reconstructed mass (up to 9.9% (95% CI: 5.2%–15.7%) for the horse mackerel). Pilchards were also still among the most abundant prey (46%), reaching a contribution to the overall diet of 23.9% (95% CI: 16.4%–32.5%) by reconstructed mass. Other common prey species were sandeels (Ammodytidae; 36% of occurrence), sprat ( *Sprattus sprattus* ; 35%) and mackerel (*Scomber* spp.; 30%), reaching respectively 5.6% (95% CI: 1.6%–12.4%), 3.6% (95% CI: 1.9%–5.9%) and 3.6% (95% CI: 2.2%–5.7%) of reconstructed mass. The presence of pout and European hake is also noteworthy, with approximately 26% of occurrence and reaching 6.1% (95% CI: 2.5%–10.1%) of reconstructed mass for hake.

Fourty‐two prey species were still present in the fresh diet. Among them, anchovy was the most occurrent (62%) and abundant (48% (95% CI: 34.9%–60.8%)) prey species. Several other species were also commonly found, such as Sepiolidae, Gobiidae (mostly *Pomatoschistus* spp.), pilchards, horse mackerel, and sprat, occurring in 30% to 40% of the samples. However, considering their relative importance by number and reconstructed mass, only pilchards (respectively 15.4% (95% CI: 9.3%–23.6%) and 41.3% (95% CI: 28.8%–53.6%)) were also major species. In terms of reconstructed mass, sandeels and mackerels were also relatively important, reaching 5.8% (95% CI: 0.6%–16.1%) and 7.8% (95% CI: 4.0%–12.8%) of total mass.

### 3.2 Diet Comparison

#### 3.2.1 Composition

**FIGURE 2:**
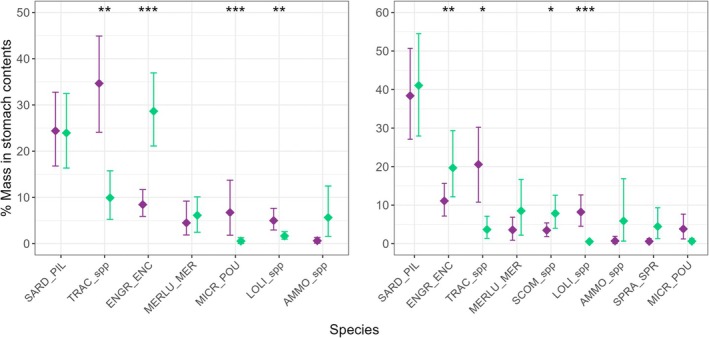
Comparison of contribution in reconstituted mass of main prey species between 1999 and 2006 (purple) and 2017–2019 (green) in total diet (left panel) and fresh diet (right panel). *p < 0.05; **p < 0.01 and ***p < 0.001. SARD_PIL: *Sardina pilchardus* ; TRAC_spp: *Trachurus* spp.; ENGR_ENC: *Engraulis encrasicolus* ; MERLU_MER: *Merluccius merluccius* ; MICR_POU: *Micromesistius poutassou* ; LOLI_spp: *Loligo* spp.; AMMO_spp: Ammodytidae; SCOM_spp: *Scomber* spp.; SPRA_SPR: *Sprattus sprattus* .

No difference in diversity was found (Table 4). Analysis of reconstructed mass composition of the total diet showed a switch in major prey from the *former* to the *recent* period, with a significant decrease in horse mackerel contribution (*t* = 2.8365, *p* < 0.01) and an increase in anchovy contribution (*t* = −4.2636, *p* < 0.01) to the overall diet while pilchard and European hake contributions did not vary (Figure 2A).

**TABLE 4:** Diversity indices of prey items identified from stomach contents of common dolphin pooled by periods in the total and the fresh diet.

		Periods	
		Former (1999–2006)	Recent (2017–2019)
Total diet	Alpha diversity	6.76 ± 2.76	6.84 ± 3.33
	Shannon	1.04 ± 0.45	0.93 ± 0.50
	Simpson	0.51 ± 0.21	0.45 ± 0.23
Fresh diet	Alpha diversity	3.5 ± 2.18	4.09 ± 2.82
	Shannon	0.63 ± 0.49	0.66 ± 0.50
	Simpson	0.34 ± 0.25	0.34 ± 0.24

**FIGURE 3:**
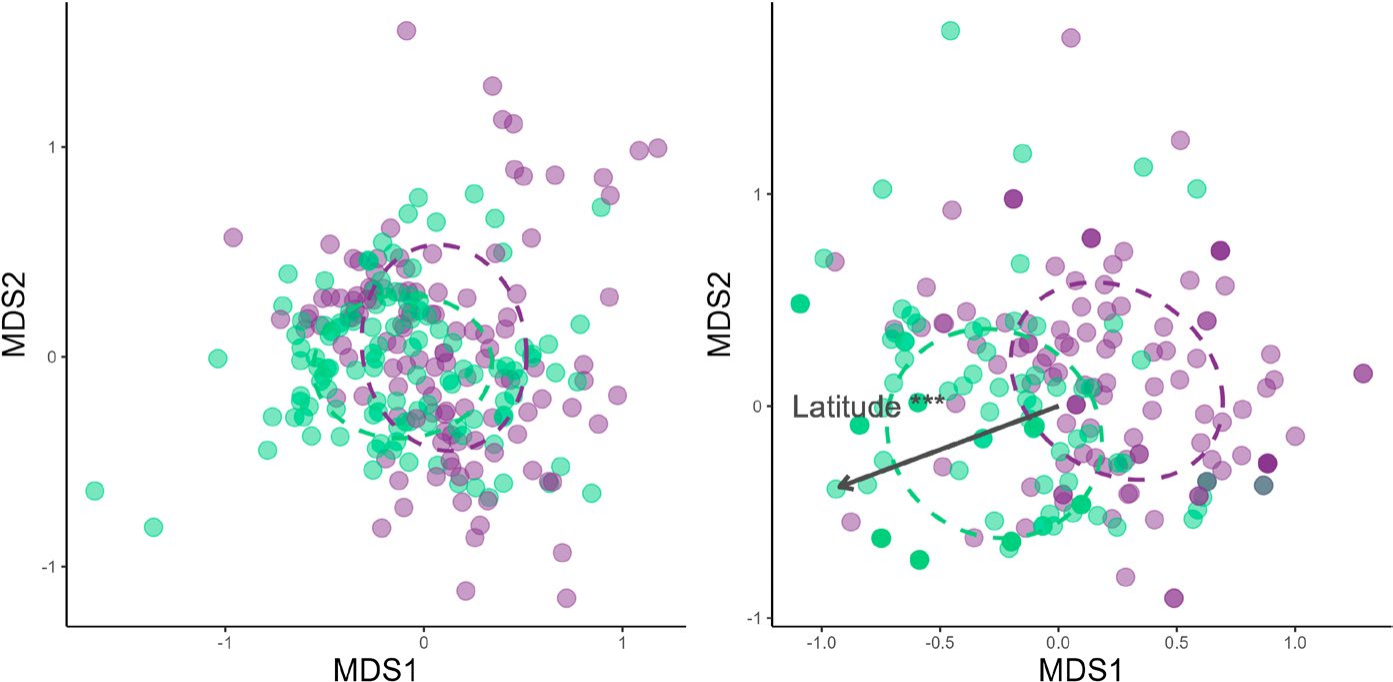
Nonmetric multidimensional scaling (nMDS) ordinations of the importance in mass of different prey species in total diet (left panel) and fresh diet (right panel) with SE ellipses based on the time period. Purple dots represent the samples of the former period and green dots, the samples of the recent period. Significant variable to explain the ordination of samples is represented with a dark arrow. ***P < 0.001.

In addition, a few other minor preys showed differences in their contribution to the diet between the two time periods. For example, the relative importance by mass of blue whiting and squids decreased (*W* = 9353, *p* < 0.01 and *t* = 2.8365, *p* < 0.01, respectively). However, nMDS ordinations and statistical analyses were unable to identify any differences in dietary composition between the two time periods (ANOSIM, global R statistic 0.057, *p* = 0.001; Figure 3A). The diet overlap analysis also indicated a high degree of overlap in the overall diet between the two periods (Pianka's Index, 0.77).

In the fresh diet, variations in the contribution of preys were similar, with a decrease in horse mackerel (*t* = 2.1549, *p* < 0.05) and squids (*t* = 3.7954, *p* < 0.01), and an increase in anchovy (*t* = −2.7289, *p* < 0.01; Figure 2B). In addition, the relative importance of mackerel and sprat increased in the recent period, but the variation was significant for mackerel only (*t* = −2.1082, *p* < 0.05). nMDS ordinations and statistical analyses for the fresh diet found a significant difference in the diet of the two time periods (ANOSIM, global R statistic 0.175, *p* < 0.001, Figure 3B). Environmental and biological variables were fitted to the nMDS ordination, and *Latitude* was found to be significant in explaining the ordination of samples (*r* ^2^ = 0.0756, *p* < 0.001) as a function of the axis MDS1, but sex and size were not significant. The diet overlap analysis, however, highlighted a fairly high degree of overlap in the fresh diet between the two periods (Pianka's Index, 0.64).

#### 3.2.2 Prey Size

**FIGURE 4:**
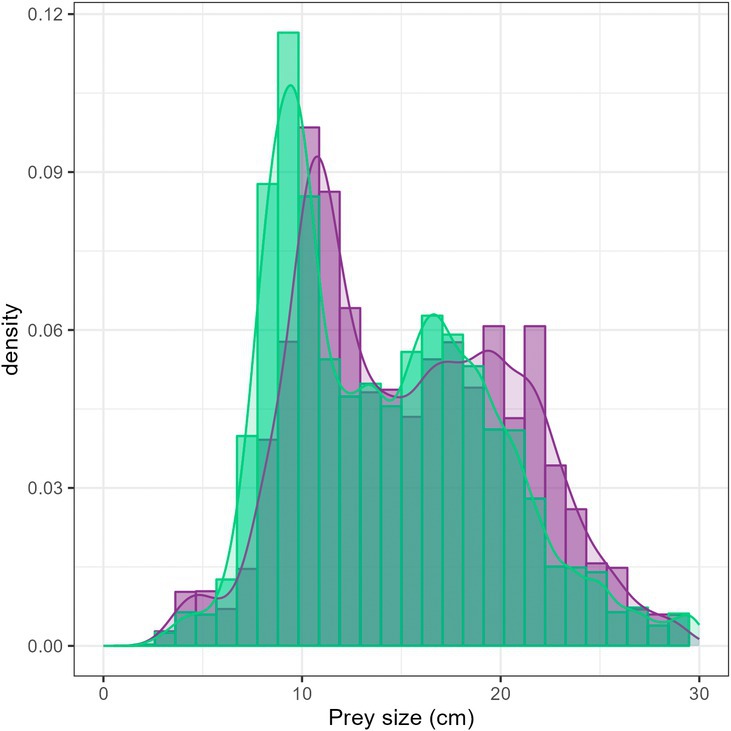
Comparison of the global fish length distribution (cm) weighted by their contribution in mass between the former period (1999–2006; purple) and the recent period (2017–2019; green).

In total, prey size did not vary depending on the time period (Figure 4). While a few larger preys occurred, such as *Boops boops* , *Clupea harengus* , *Conger conger* , and *Solea* spp., the size of some important prey increased (Table 5).

**TABLE 5:** Individual body size of fish species, number of specimens, mean of fish length (FL in cm) and body mass (BM in g) *±* standard deviation and ranges of value for each period (former and recent).

Prey species	Individual body size									
	Former period (1999–2006)					Recent period (2017–2019)				
	*N*	FL ± SD	Range	BM ± SD	Range	*N*	FL ± SD	Range	BM ± SD	Range
Fish										
Caproidae										
*Capros aper*	—	—	—	—	—	8	5.2 ± 0.6	[4.2–5.9]	3.3 ± 0.9	[1.9–4.6]
Congridae										
*Conger conger*	—	—	—	—	—	7	39.7 ± 13.7	[10.0–48.6]	188.9 ± 101.2	[1.2–286.1]
Argentinidae										
*Argentina* spp.	139	13.3 ± 3.6	[7.3–20.9]	15.6 ± 12.3	[1.6–54.9]	95	8.4 ± 13.4	[6.0–10.9]	2.9 ± 1.5	[0.9–6.2]
Bathylagidae										
*Unidentified Bathylagidae*	22	10.4 ± 1.2	[8.4–12.6]	96.9 ± 37.9	[45.6–181.2]	—	—	—	—	—
Atherinidae										
*Atherina presbyter*	48	9.2 ± 2.5	[3.8–13.1]	6.7 ± 4.4	[0.4–16.5]	166	9.0 ± 2.4	[4.1–15.1]	6.5 ± 5.6	[0.5–25.5]
Belonidae										
*Belone belone*	7	59.6 ± 6.3	[54.2–72.7]	279.4 ± 100.4	[203.5–495.7]	12	51.1 ± 7.7	[43.4–66.6]	181.5 ± 93.3	[103.4–378.9]
Callionymidae										
*Callionymus* spp.	—	—	—	—	—	3	5.8 ± 3.0	[3.9–9.2]	2.2 ± 2.9	[0.4–5.6]
Carangidae										
*Trachurus* spp.	2200	9.2 ± 3.7	[2.9–35.6]	13.2 ± 25.8	[0.3–396.7]	918	8.6 ± 2.9	[3.6–27.3]	9.9 ± 16.8	[0.6–188.6]
Alosidae										
*Sardina pilchardus*	1276	18.3 ± 3.5	[7.2–30.2]	56.1 ± 29.9	[3.0–229.0]	988	15.9 ± 3.3	[7.2–23.6]	37.7 ± 21.7	[3.0–109.2]
Clupleidae										
*Clupea harengus*	—	—	—	—	—	4	22.6 ± 6.4	[16.3–28.2]	86.2 ± 63.8	[26.8–142.5]
*Sprattus sprattus*	74	11.4 ± 2.4	[6.2–17.4]	11.5 ± 6.5	[1.6–36.5]	625	10.2 ± 2.1	[4.1–16.0]	8.0 ± 4.8	[0.4–28.2]
Engraulidae										
*Engraulis encrasicolus*	2077	11.3 ± 2.3	[5.1–18.5]	10.3 ± 6.4	[0.8–39.2]	2097	9.0 ± 1.7	[4.1–21.8]	5.0 ± 3.8	[0.4–63.5]
*Unidentified Clupeiformes*	5	20.3 ± 2.2	[17.7–22.6]	72.0 ± 22.8	[45.8–95.6]	—	—	—	—	—
Labridae										
*Unidentified Labridae*	2	8.9 ± 0.7	[8.4–9.4]	5.9 ± 2.0	[4.5–7.3]	39	10.0 ± 3.1	[6.0–17.3]	14.7 ± 20.5	[1.1–71.5]
Sparidae										
*Boops boops*	—	—	—	—	—	3	25.8 ± 1.7	[23.9–26.8]	162.0 ± 30.3	[126.9–180.3]
Sparidae spp.	6	11.1 ± 3.5	[6.5–15.0]	25.5 ± 19.3	[3.6–52.0]	2	8.6 ± 0.02	[8.6–8.6]	9.1 ± 0.06	[9.0–9.1]
Gadidae										
*Gadiculus argenteus*	10	4.6 ± 1.2	[3.2–6.5]	1.2 ± 1.2	[0.2–3.5]	61	7.0 ± 0.8	[5.3–9.0]	5.2 ± 2.7	[1.5–14.1]
*Merlangius merlangus*	66	16.0 ± 4.1	[8.7–28.0]	39.0 ± 28.4	[4.9–182.8]	70	19.5 ± 5.6	[6.3–33.4]	77.2 ± 71.7	[1.8–318.0]
*Micromesistius poutassou*	506	13.3 ± 3.8	[5.8–26.3]	20.2 ± 19.1	[1.3–124.1]	108	18.6 ± 4.9	[7.0–31.8]	54.1 ± 48.2	[5.7–219.6]
*Trisopterus* spp.	534	9.3 ± 4.6	[1.6–28.9]	16.3 ± 27.0	[0.07–229.6]	307	8.4 ± 4.2	[0.8–20.2]	11.7 ± 14.7	[0.01–84.1]
Unidentified Gadidae	30	6.8 ± 1.1	[4.9–9.2]	2.4 ± 1.1	[0.8–5.4]	—	—	—	—	—
Lotidae										
*Molva molva*	2	36.4 ± 6.0	[32.2–40.6]	258.7 ± 128.5	[167.9–349.6]	—	—	—	—	—
Gaidropsaridae (Rockling)										
Unidentified Gaidropsaridae	110	12.6 ± 3.7	[6.8–28.6]	8.9 ± 9.0	[1.6–61.4]	44	11.2 ± 1.4	[9.0–14.5]	5.8 ± 1.9	[3.2–10.8]
Merlucciidae										
*Merluccius merluccius*	319	14.5 ± 5.9	[4.8–37.9]	33.9 ± 47.7	[0.8–383.4]	359	16.1 ± 6.7	[4.4–36.5]	47.0 ± 59.8	[0.7–342.7]
Gobiidae						1244	4.3 ± 1.0	[1.5–7.4]	0.7 ± 0.5	[0.02–3.5]
*Aphia minuta*	—	—	—	—	—	346	5.0 ± 0.8	[2.7–7.4]	0.9 ± 0.4	[0.1–2.9]
*Gobius* spp.	—	—	—	—	—	2	6.5 ± 0.2	[6.3–6.7]	3.2 ± 0.3	[3.0–3.5]
*Lesueurigobius friesii*	—	—	—	—	—	102	4.5 ± 1.1	[2.3–6.8]	0.9 ± 0.6	[0.1–2.6]
*Pomatoschistus* spp.	—	—	—	—	—	508	4.2 ± 0.8	[2.1–7.3]	0.7 ± 0.5	[0.1–3.5]
Unidentified Gobiidae	2465	4.1 ± 1.0	[1.5–12.6]	0.7 ± 0.9	[0.02–19.4]	286	3.3 ± 0.7	[1.4–4.8]	0.3 ± 0.2	[0.02–0.9]
Mugilidae										
Unidentified Mugilidae	1	30.2		366.1		52	13.2 ± 3.2	[8.4–24.9]	38.4 ± 29.3	[6.5–201.0]
Mullidae										
*Mullus surmuletus*	—	—	—	—	—	6	11.4 ± 1.8	[9.6–13.2]	18.6 ± 8.8	[9.9–28.0]
Myctophidae										
*Benthosema glaciale*	2	3.7 ± 0.1	[3.6–3.7]	0.5 ± 0.02	[0.4–0.5]	42	4.3 ± 1.0	[2.0–5.9]	0.9 ± 0.5	[0.1–1.9]
*Ceratoscopelus maderensis*						1	4	—	0.8	—
*Myctophum punctatum*	16	6.1 ± 0.9	[5.2–8.8]	2.0 ± 1.2	[1.1–6.1]	38	5.9 ± 1.0	[4.0–8.1]	1.8 ± 1.0	[0.5–4.6]
*Notoscopelus kroeyeri*	6	4.8 ± 0.2	[4.6–5.0]	1.2 ± 0.1	[1.0–1.3]	20	5.6 ± 1.4	[3.3–8.9]	2.3 ± 1.8	[0.3–8.2]
Ammodytidae										
Unidentified Ammodytidae	137	13.6 ± 4.0	[6.3–28.9]	12.9 ± 11.7	[1.4–80.3]	511	12.3 ± 4.9	[3.7–33.6]	11.6 ± 15.9	[0.3–120.1]
Flatfish										
Bothidae										
*Arnoglossus* spp.	—	—	—	—	—	18	7.3 ± 1.8	[5.0–11.2]	3.5 ± 2.9	[0.9–10.8]
Unidentified Bothidae	—	—	—	—	—	4	8.8 ± 2.2	[6.8–10.9]	8.9 ± 6.0	[3.6–15.1]
Pleuronectidae										
Unidentified Pleuronectidae	12	12.2 ± 1.3	[9.4–15.2]	15.2 ± 5.3	[6.3–29.2]	—	—	—	—	—
Scophthalmidae										
Unidentified Scophthalmidae	—	—	—	—	—	11	7.1 ± 0.7	[6.2–8.2]	4.2 ± 1.2	[2.7–6.2]
Soleidae										
*Dicologlossa cuneata*	2	8.2 ± 1.1	[7.5–9.0]	7.3 ± 2.2	[5.8–8.9]	—	—	—	—	—
*Microchirus* spp. /*Buglossidium* spp.	4	12.7 ± 5.1	[8.0–17.2]	31.3 ± 29.4	[5.2–57.1]	17	12.2 ± 3.2	[7.0–18.5]	23.9 ± 20.2	[3.4–71.4]
*Solea* spp.	—	—	—	—	—	38	16.1 ± 4.0	[9.5–25.7]	40.1 ± 31.8	[5.8–150.2]
Scombridae										
*Scomber* spp.	97	17.5 ± 5.9	[8.6–36.4]	70.4 ± 89.5	[4.8–518.7]	157	17.4 ± 4.1	[9.5–26.2]	55.9 ± 40.2	[6.4–178.3]
Sternoptychidae										
*Maurolicus muelleri*	—	—	—	—	—	99	2.6 ± 0.05	[2.5–2.7]	0.2 ± 0.01	[0.2–0.3]

**FIGURE 5:**
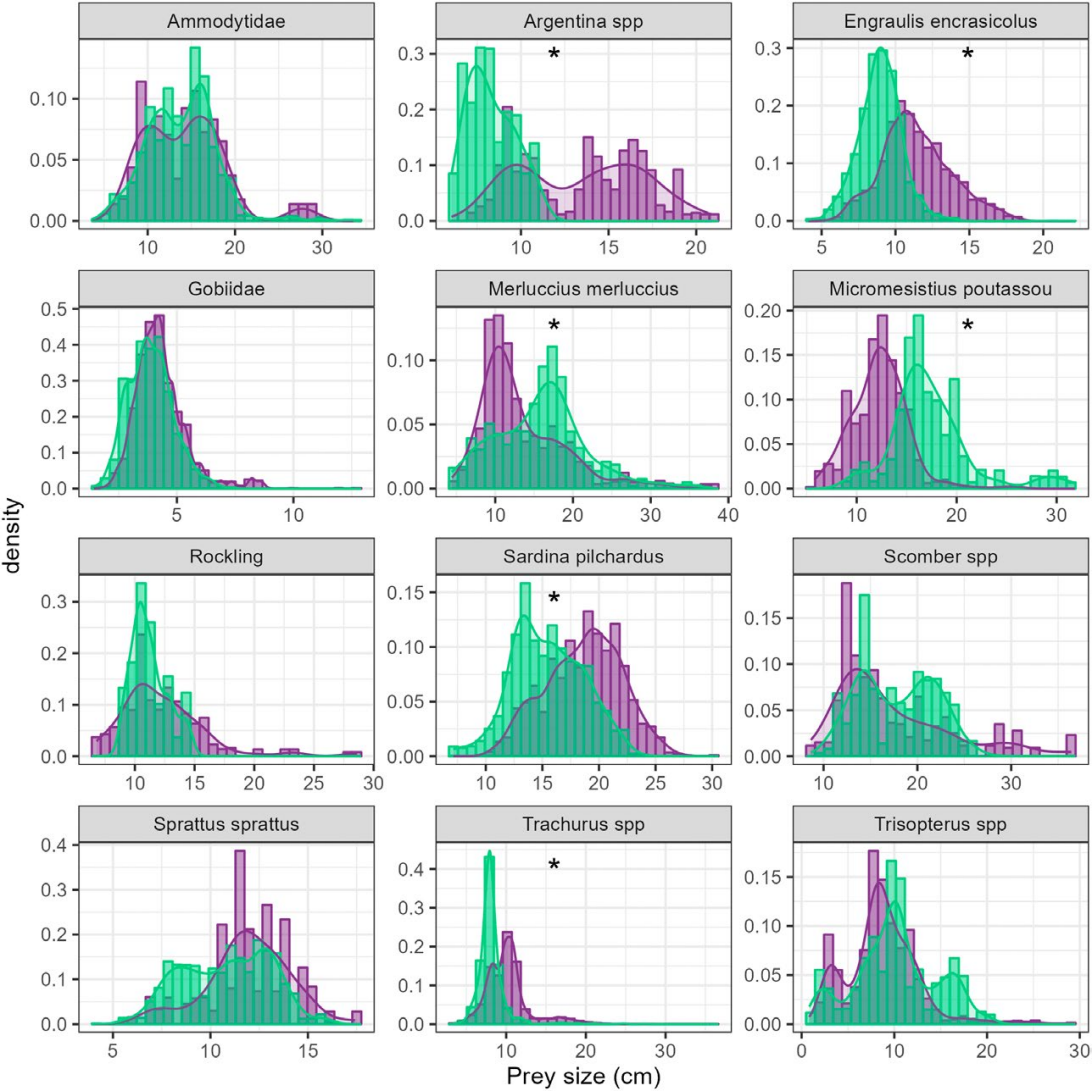
Comparison of fish length distributions (cm) for main species weighted by their abundance between the former period (purple) and the recent period (green). *p < 0.001.

For instance, it is the case for European hake with an increase of 2.3 cm TL of the mean size (*t* = 4.58, *p* < 0.001) and blue whiting (+5.1 cm TL, *t* = 11.55, *p* < 0.001; Figure 5). However, between the two time periods, the size of the most important preys in terms of contribution decreased, such as for pilchards (−3.2 cm TL, *t* = −19.178, *p* < 0.001), anchovy (−2.5 cm TL, *t* = −31.817, *p* < 0.001), horse mackerel (−2.0 cm TL, *t* = −19.619, *p* < 0.001) and argentines (−5.1 cm TL, *t* = −13.661, *p* < 0.001). For some other preys such as sandeels, whiting, mackerel, and sprat, no differences in size were found.

## 4 Discussion

The present study provides new information on the diet of the short‐beaked common dolphin in the Bay of Biscay between 1999 and 2019. Although the overall diet of the common dolphin was still mainly composed of small schooling energy‐rich fish, we revealed some significant variations in the biomass contributions of different prey species over time. Additionally, a decrease in the mean size of some main prey was also found. Furthermore, the predominance of pilchard and anchovy in the fresh remains of bycatch individuals, whatever the period studied, suggests that foraging for these prey species resulted in a higher risk of bycatch. While some limitations may exist regarding estimations of reconstructed biomass of prey using allometric regressions and potential under‐ or over‐estimations of some prey because of different digestion rates (Amundsen and Sánchez‐Hernández 2019; Buckland et al. 2017; Wijnsma et al. 1999), relative comparison of results is still valuable. In addition, the number of samples and the number of prey retrieved in stomach contents provide robustness to our results, while acknowledging the potential sampling bias. Further studies using ecological tracers, such as stable isotope analyses, could therefore be conducted to complement our results and provide a broader vision on a larger scale of integration (Crawford et al. 2008; Fontaine et al. 2007; Monteiro et al. 2015).

### 4.1 Dietary Stability of the Common Dolphin (1999–2019)

In this study, we found no evidence of variations in the prey type preferences of common dolphins captured as bycatch in the Bay of Biscay in winter, which remained mainly composed of high‐energy density prey species (Meynier et al. 2008; Spitz et al. 2014). This aligns with existing knowledge about the common dolphin's feeding strategy, which involves quality‐based selection of prey to meet their energetically expensive lifestyle (Spitz et al. 2012, 2010).

However, we observed specific variations within energy‐rich species. For instance, there was a significant decrease in the relative importance of horse mackerel over time and a corresponding increase in anchovy. This can be explained by the recent collapse of the horse mackerel population, whereas anchovy biomass increased following the fishery closure that occurred between 2000 and 2005 in the Bay of Biscay (Doray, Petitgas, et al. 2018; ICES 2024). The diet of a predator is indeed influenced by multiple interacting factors, including prey abundance. According to optimal foraging theory, a predator ranks available prey types based on their energetic profitability (i.e., calorie content divided by handling time), thereby maximizing energy intake to foraging time and associated energy costs. Such variations have already been observed in the common dolphin in the Bay of Biscay and Iberian waters and were related to prey abundance changes (Meynier et al. 2008; Santos et al. 2013). The ecosystem of the Bay of Biscay has changed and will continue to change due to climate and human pressures affecting particularly with regard to oceanographic conditions, as well as the dynamics and composition of prey populations (Chust et al. 2011; Marchand et al. 2020) Specifically, pilchard and anchovy spawning stock biomass (SSB) have known important fluctuations in the last decades; their recruitments are affected by climatic factors (e.g., upwelling intensity and wind circulation; Borja et al. 2008) and could be changing in the coming years; hence affecting food availability for their predators, including the common dolphin.

### 4.2 Toward a Spatial Shift to the Coast?

Regarding minor species, few changes were observed over time. Notably, the importance of blue whiting, pouts, and squids decreased in favor of sandeels and sprats. Blue whiting and squids are more abundant prey in outer shelf and upper slope habitats, while sandeels and sprats are more related to coastal waters (Doray, Hervy, et al. 2018). These changes could be related to changes in the spatial distribution of dolphins and/or shifts in the spatial distribution of major prey toward more coastal waters (Laran et al. 2022), thus increasing the spatial overlap between dolphins and the main fishing effort in the Bay of Biscay, which is concentrated near the coast (Demanèche et al. 2021).

Although no difference was found in the overall size spectrum of prey between the two time periods, there were specific differences in size for the main prey of the diet. This was particularly significant for pilchards, anchovy, and horse mackerel, which are among the most energy‐rich species. Changes in size and body condition in the environment had already been identified for such species, particularly for pilchards (Chust et al. 2022; Doray, Petitgas, et al. 2018; Véron et al. 2020) and European anchovy (Chust et al. 2022; Taboada et al. 2024), suggesting that the decrease in size observed in the diet is related to environmental variations. Such variations in prey size for common dolphins were already observed in Portuguese waters with the disappearance of smaller sizes of pilchards in common dolphin diet related to poor recruitment to the stock (Marçalo et al. 2018). In the Bay of Biscay, it has also been found that the average size of small pelagic fishes varied depending on spatial distribution. In particular, there is a decrease in mean body size when approaching the coast (Doray, Hervy, et al. 2018), hence supporting the hypothesis of dolphins foraging closer to shore. However, the reduction in body size of small pelagic fish is also commonly attributed to ocean warming (Taboada et al. 2024).

### 4.3 Dietary Habits and Potential Link to Bycatch Events

Stomach contents usually include fresh and accumulated remains representing a time of integration of several food intakes. By considering only the fresh fraction, we reduced the integration time to the last food intake, focusing on a finer temporal scale close to death. In this study, the last food intake can inform on preys associated with the bycatch event. In addition, focusing on the fresh fraction only allowed us to mitigate the potential bias associated with varied digestion time from one prey to another that can lead to a misestimation of some species (Brown et al. 2012). We found a majority (95% for the recent period) of the stomachs sampled contained fresh prey, indicating that the majority of the animals studied died during or shortly after feeding, which suggests that dolphins are being caught while feeding.

The diet profile of the fresh fraction (diversity of prey and their contribution to the diet) was consistent with the overall diet, mostly composed of small pelagic fish. This finding suggests there is no specific feeding strategy leading to bycatch, such as depredation, contrary to what is observed in Iberian waters, for example. Common dolphins are indeed regularly interacting with purse seine fisheries targeting small pelagic fish species, suggesting that dolphins would take advantage of the fishery to forage, eventually leading to bycatch (Marçalo et al. 2018).

In the Bay of Biscay, fresh pilchards, horse mackerels, and anchovies dominated the prey species targeted during the bycatch event, with > 60% of the ingested biomass. At least one of these species was found in more than 80% of the animals studied, while Sepiolidae, mackerel, and *Loligo* spp. were the most occurrent species in the remaining 20%. These species are not targeted by fisheries in which dolphins are captured (mostly targeting large demersal species, such as sea bass). There is, however, one exception with the European hake, which is both targeted by fisheries in which dolphins are caught as bycatch and by dolphins. The targeted sizes are different, though. The minimum marketable size of European hake in Europe (e.g., North‐East Atlantic: 27 cm, JORF n°0045 2013; https://www.legifrance.gouv.fr/eli/arrete/2013/1/29/TRAM1240353A/jo/texte) is indeed almost twice as big as the size of hake targeted by dolphins (from 14.5 ± 5.9 cm to 16.1 ± 6.7 cm on average, respectively for the former and recent periods; Table 5). These results therefore suggest that dolphins are not caught as bycatch when foraging on commercial species.

Trophic overlap between common dolphins and some commercial fish species, such as sea bass or hake, could rather be explained bycatch. Notably, in pelagic trawls, both dolphins and targeted commercial fish species could simultaneously forage on small pelagic fish (Corrales et al. 2022; Spitz et al. 2013). Furthermore, small pelagic fish species are also part of the diet of the anglerfish ( *Lophius piscatorius* ), in the northern Atlantic Ocean, an important species targeted by fisheries at risk for dolphin bycatch (gillnets and trammel nets; Paillé et al. 2024). Issac et al. (2017) suggested that pelagic species such as horse mackerel and mackerel could be ingested when they approach the bottom. One must assume that dolphins would also forage on small pelagic fish close to the bottom rather than in the upper part of the column water. This can also explain the presence of a few benthic preys in their diet, such as flatfish. Bycatch events would therefore be more linked to spatial and temporal overlap between dolphins and their prey on fishing grounds rather than direct foraging associations between dolphins, fishing gears, and targeted commercial fish species.

## 5 Conclusion

This study provides a comprehensive analysis of the diet of the short‐beaked common dolphin in the Bay of Biscay over two decades (1999–2019). The findings reveal that while the overall diet remains composed mainly of high‐energy prey species, there are notable temporal variations in prey composition and size. Specifically, we observed significant shifts in the prevalence of certain species, such as a decrease in horse mackerel and an increase in anchovy, which correspond to changes in their environmental abundance. The analysis of the fresh fraction has proven to be crucial in understanding the immediate dietary patterns linked to bycatch events. The consistency between the fresh fraction and overall diet suggests that the bycatch events do not occur because of a feeding behavior that differs from the general habits of the species, but three prey species nevertheless seem to be more involved in the capture phenomenon. Among the variety of prey species included in the winter diet of the common dolphin, feeding on anchovies, pilchards, and horse mackerel appears to increase their risk of capture. Finally, the recent decrease in prey size for these species could also increase their predation efforts and the duration of feeding activities. This, in turn, may elevate their risk of encountering fishing gear, thereby increasing bycatch incidents.

## Author Contributions

**Johanna Faure:** conceptualization (equal), formal analysis (lead), methodology (equal), visualization (lead), writing – original draft (lead). **Jasmin Niol:** investigation (equal). **Eléonore Meheust:** data curation (lead). **Jérôme Spitz:** conceptualization (equal), funding acquisition (lead), investigation (lead), methodology (equal), supervision (lead), writing – review and editing (lead).

## Conflicts of Interest

The authors declare no conflicts of interest.

## Supporting information


Appendix S1.

## Data Availability

The data that support the findings of this study will be openly available through the data. InDoRES platform at https://doi.org/10.48579/PRO/YVJZ3A.

## References

[ece371815-bib-0001] Allain, G. , P. Petitgas , and P. Lazure . 2001. “The Influence of Mesoscale Ocean Processes on Anchovy ( *Engraulis encrasicolus* ) Recruitment in the Bay of Biscay Estimated With a Three‐Dimensional Hydrodynamic Mode.” Fisheries Oceanography 10: 151–163. 10.1046/j.1365-2419.2001.00164.x.

[ece371815-bib-0002] Amundsen, P.‐A. , and J. Sánchez‐Hernández . 2019. “Feeding Studies Take Guts – Critical Review and Recommendations of Methods for Stomach Contents Analysis in Fish.” Journal of Fish Biology 95: 1364–1373. 10.1111/jfb.14151. 31589769

[ece371815-bib-0003] Avila, I. C. , K. Kaschner , and C. F. Dormann . 2018. “Current Global Risks to Marine Mammals: Taking Stock of the Threats.” Biological Conservation 221: 44–58. 10.1016/j.biocon.2018.02.021.

[ece371815-bib-0004] Baird, R. W. , D. B. Anderson , M. A. Kratofil , and D. L. Webster . 2021. “Bringing the Right Fishermen to the Table: Indices of Overlap Between Endangered False Killer Whales and Nearshore Fisheries in Hawai'i.” Biological Conservation 255: 108975. 10.1016/j.biocon.2021.108975.

[ece371815-bib-0005] Barbraud, C. , G. N. Tuck , R. Thomson , K. Delord , and H. Weimerskirch . 2013. “Fisheries Bycatch as an Inadvertent Human‐Induced Evolutionary Mechanism.” PLoS One 8: e60353. 10.1371/journal.pone.0060353. 23593199 PMC3622665

[ece371815-bib-0006] Borja, A. , D. Amouroux , P. Anschutz , M. Gómez‐Gesteira , M. C. Uyarra , and L. Valdés . 2019. “The Bay of Biscay.” In World Seas: An Environmental Evaluation, 113–152. Elsevier. 10.1016/B978-0-12-805068-2.00006-1.

[ece371815-bib-0007] Borja, A. , A. Fontán , J. Sáenz , and V. Valencia . 2008. “Climate, Oceanography, and Recruitment: The Case of the Bay of Biscay Anchovy ( *Engraulis encrasicolus* ).” Fisheries Oceanography 17: 477–493. 10.1111/j.1365-2419.2008.00494.x.

[ece371815-bib-0008] Bowen, W. D. , and S. J. Iverson . 2013. “Methods of Estimating Marine Mammal Diets: A Review of Validation Experiments and Sources of Bias and Uncertainty.” Marine Mammal Science 29: 719–754. 10.1111/j.1748-7692.2012.00604.x.

[ece371815-bib-0009] Brown, S. C. , J. J. Bizzarro , G. M. Cailliet , and D. A. Ebert . 2012. “Breaking With Tradition: Redefining Measures for Diet Description With a Case Study of the Aleutian Skate *Bathyraja aleutica* (Gilbert 1896).” Environmental Biology of Fishes 95: 3–20. 10.1007/s10641-011-9959-z.

[ece371815-bib-0010] Buckland, A. , R. Baker , N. Loneragan , and M. Sheaves . 2017. “Standardising Fish Stomach Content Analysis: The Importance of Prey Condition.” Fisheries Research 196: 126–140. 10.1016/j.fishres.2017.08.003.

[ece371815-bib-0011] Castro, J. , L. Cañás , J. Rodríguez , G. J. Pierce , and C. Saavedra . 2024. “An Evaluation of the Cetacean Bycatch Monitoring Programme on Board the Spanish Set Gillnet and Bottom Pair Trawl Fleets in the Bay of Biscay.” ICES Journal of Marine Science 81: 307–316. 10.1093/icesjms/fsad197.

[ece371815-bib-0012] Chust, G. , Á. Borja , A. Caballero , et al. 2011. “Climate Change Impacts on Coastal and Pelagic Environments in the Southeastern Bay of Biscay.” Climate Research 48: 307–332. 10.3354/cr00914.

[ece371815-bib-0013] Chust, G. , M. González , A. Fontán , et al. 2022. “Climate Regime Shifts and Biodiversity Redistribution in the Bay of Biscay.” Science Total Environment 803: 149622. 10.1016/j.scitotenv.2021.149622. 34496346

[ece371815-bib-1002] Clarke, M. R. 1986. A Handbook for the Identification of Cephalopod Beaks. Clarendon Press, Oxford University Press. http://ci.nii.ac.jp/ncid/BA01136737.

[ece371815-bib-0014] Corrales, X. , I. Preciado , D. Gascuel , et al. 2022. “Structure and Functioning of the Bay of Biscay Ecosystem: A Trophic Modelling Approach.” Estuarine, Coastal and Shelf Science 264: 107658. 10.1016/j.ecss.2021.107658.

[ece371815-bib-0015] Cortés, E. 1997. “A Critical Review of Methods of Studying Fish Feeding Based on Analysis of Stomach Contents: Application to Elasmobranch Fishes.” Canadian Journal of Fisheries and Aquatic Sciences 54: 13.

[ece371815-bib-0016] Crawford, K. , R. A. Mcdonald , and S. Bearhop . 2008. “Applications of Stable Isotope Techniques to the Ecology of Mammals.” Mammal Review 38: 87–107. 10.1111/j.1365-2907.2008.00120.x.

[ece371815-bib-0017] Crowder, L. B. , E. L. Hazen , N. Avissar , R. Bjorkland , C. Latanich , and M. B. Ogburn . 2008. “The Impacts of Fisheries on Marine Ecosystems and the Transition to Ecosystem‐Based Management.” Annual Review of Ecology, Evolution, and Systematics 39: 259–278.

[ece371815-bib-0018] Davidson, A. D. , A. G. Boyer , H. Kim , et al. 2012. “Drivers and Hotspots of Extinction Risk in Marine Mammals.” Proceedings of the National Academy of Sciences of the United States of America 9: 3395–3400. 10.1073/pnas.1121469109. PMC3295301 22308490

[ece371815-bib-0019] Demanèche, S. , P. Berthou , A. Biseau , et al. 2021. Caractérisation et typologie des engins et de l'effort de pêche dans le Golfe de Gascogne en période d'échouage des petits cétacés. DPMA – Direction des Pêches Maritimes et de l'Aquaculture, La Défense.

[ece371815-bib-0020] Demanèche, S. , P. Berthou , S. Le Blond , et al. 2019. Amélioration de la connaissance de l'activité des fileyeurs dans le golfe de Gascogne. https://archimer.ifremer.fr/doc/00506/61725/65698.pdf.

[ece371815-bib-0021] Demaster, D. P. , C. W. Fowler , S. L. Perry , and M. F. Richlen . 2001. “Predation and Competition: The Impact of Fisheries on Marine‐Mammal Populations Over the Next One Hundred Years.” Journal of Mammalogy 82: 641–651.

[ece371815-bib-0022] Doray, M. , C. Hervy , M. Huret , and P. Petitgas . 2018. “Spring Habitats of Small Pelagic Fish Communities in the Bay of Biscay.” Progress in Oceanography Multidisciplinary integrated surveys 166: 88–108. 10.1016/j.pocean.2017.11.003.

[ece371815-bib-0023] Doray, M. , P. Petitgas , M. Huret , et al. 2018. “Monitoring Small Pelagic Fish in the Bay of Biscay Ecosystem, Using Indicators From an Integrated Survey.” Progress in Oceanography 166: 168–188. 10.1016/j.pocean.2017.12.004.

[ece371815-bib-0024] Dulvy, N. K. , S. L. Fowler , J. A. Musick , et al. 2014. “Extinction Risk and Conservation of the World's Sharks and Rays.” eLife 3: e00590. 10.7554/eLife.00590. 24448405 PMC3897121

[ece371815-bib-0025] Ferro de Godoy, D. , J. T. Mendonça , and A. Andriolo . 2020. “Occurrence of Guiana Dolphin ( *Sotalia guianensis* ) in Southeast of Brazil: Driven by Prey Distribution or Human Fishing Activity?” Aquatic Conservation: Marine and Freshwater Ecosystems 30: 1910–1921. 10.1002/aqc.3367.

[ece371815-bib-0026] Fontaine, M. C. , K. A. Tolley , U. Siebert , et al. 2007. “Long‐Term Feeding Ecology and Habitat Use in Harbour Porpoises *Phocoena phocoena* From Scandinavian Waters Inferred From Trace Elements and Stable Isotopes.” BMC Ecology 7: 1. 10.1186/1472-6785-7-1. 17229317 PMC1781931

[ece371815-bib-0027] Furness, R. W. 2003. “Impacts of Fisheries on Seabird Communities.” Scientia Marina 67: 33–45. 10.3989/scimar.2003.67s233.

[ece371815-bib-0028] Gilles, A. , M. Authier , N. Ramirez‐Martinez , et al. 2023. Estimates of Cetacean Abundance in European Atlantic Waters in Summer 2022 from the SCANS‐IV Aerial and Shipboard Surveys (Final Report).

[ece371815-bib-0029] Gilman, E. L. , M. Hall , H. Booth , et al. 2022. “A Decision Support Tool for Integrated Fisheries Bycatch Management.” Reviews in Fish Biology and Fisheries 32: 441–472. 10.1007/s11160-021-09693-5.

[ece371815-bib-0030] Goñi, R. 1998. “Ecosystem Effects of Marine Fisheries: An Overview.” Ocean and Coastal Management 40: 37–64. 10.1016/S0964-5691(98)00037-4.

[ece371815-bib-0031] Guénette, S. , and D. Gascuel . 2012. “Shifting Baselines in European Fisheries: The Case of the Celtic Sea and Bay of Biscay.” Ocean and Coastal Management 70: 10–21. 10.1016/j.ocecoaman.2012.06.010.

[ece371815-bib-1003] Härkönen, T. 1986. Guide to the Otoliths of the Bony Fishes of the Northeast Atlantic. Danbiu ApS.

[ece371815-bib-0032] Hazen, E. L. , K. L. Scales , S. M. Maxwell , et al. 2018. “A Dynamic Ocean Management Tool to Reduce Bycatch and Support Sustainable Fisheries.” Science Advances 4: eaar3001. 10.1126/sciadv.aar3001. 29854945 PMC5976278

[ece371815-bib-0033] Heino, M. , B. Díaz Pauli , and U. Dieckmann . 2015. “Fisheries‐Induced Evolution.” Annual Review of Ecology, Evolution, and Systematics 46: 461–480. 10.1146/annurev-ecolsys-112414-054339.

[ece371815-bib-0034] Hsieh, C. , C. S. Reiss , J. R. Hunter , J. R. Beddington , R. M. May , and G. Sugihara . 2006. “Fishing Elevates Variability in the Abundance of Exploited Species.” Nature 443: 859–862. 10.1038/nature05232. 17051218

[ece371815-bib-0035] Hutchings, J. A. , R. A. Myers , V. B. García , L. O. Lucifora , and A. Kuparinen . 2012. “Life‐History Correlates of Extinction Risk and Recovery Potential.” Ecological Applications 22: 1061–1067. 10.1890/11-1313.1. 22827118

[ece371815-bib-0036] ICES . 2024. “Working Group on Southern Horse Mackerel, Anchovy and Sardine (WGHANSA) (report).” ICES Scientific Reports. 10.17895/ices.pub.26003356.v2.

[ece371815-bib-1004] Issac, P. , M. Robert , H. Le Bris , J. Rault , L. Pawlowski , and D. Kopp . 2017. “Investigating Feeding Ecology of Two Anglerfish Species, *Lophius piscatorius* and *Lophius budegassa* in the Celtic Sea Using Gut Content and Isotopic Analyses.” Food Webs 13: 33–37.

[ece371815-bib-0037] Jennings, S. , and M. J. Kaiser . 1998. “The Effects of Fishing on Marine Ecosystems.” In Advances in Marine Biology, 201–352. Elsevier. 10.1016/S0065-2881(08)60212-6.

[ece371815-bib-0038] Jog, K. , D. Sutaria , A. Diedrich , A. Grech , and H. Marsh . 2022. “Marine Mammal Interactions With Fisheries: Review of Research and Management Trends Across Commercial and Small‐Scale Fisheries.” Frontiers in Marine Science 9: 758013. 10.3389/fmars.2022.758013.

[ece371815-bib-0039] JORF n°0045 . 2013. Arrêté du 29 janvier 2013 modifiant l'arrêté du 26 octobre 2012 déterminant la taille minimale ou le poids minimal de capture des poissons et autres organismes marins (pour une espèce donnée ou pour une zone géographique donnée) effectuée dans le cadre de la pêche maritime de loisir, NOR: TRAM1240353A.

[ece371815-bib-0040] Laran, S. , M. Authier , A. Blanck , et al. 2017. “Seasonal Distribution and Abundance of Cetaceans Within French Waters – Part II: The Bay of Biscay and the English Channel. Deep Sea Res. Part II Top.” Abundance, Distribution and Habitats of Atlantic and Mediterranean Marine Megafauna 141: 31–40. 10.1016/j.dsr2.2016.12.012.

[ece371815-bib-0041] Laran, S. , M. Genu , M. Authier , et al. 2022. Distribution et abondance de la mégafaune marine en France métropolitaine. (Rapport final de la campagne SAMM II Atlantique‐Manche – Hiver 2021). Observatoire Pelagis pour la Direction de l'Eau et de la Biodiversité et l'Office Français de la Biodiversité.

[ece371815-bib-0042] Lewison, R. L. , L. B. Crowder , A. J. Read , and S. A. Freeman . 2004. “Understanding Impacts of Fisheries Bycatch on Marine Megafauna.” Trends in Ecology & Evolution 19: 598–604. 10.1016/j.tree.2004.09.004.

[ece371815-bib-0043] Lewison, R. L. , L. B. Crowder , B. P. Wallace , et al. 2014. “Global Patterns of Marine Mammal, Seabird, and Sea Turtle Bycatch Reveal Taxa‐Specific and Cumulative Megafauna Hotspots.” Proceedings of the National Academy of Sciences 111: 5271–5276. 10.1073/pnas.1318960111. PMC3986184 24639512

[ece371815-bib-0044] Lumley, T. 2004. “Analysis of Complex Survey Samples.” Journal of Statistical Software 9: 1–19. 10.18637/jss.v009.i08.

[ece371815-bib-0045] Marçalo, A. , L. Nicolau , J. Giménez , et al. 2018. “Feeding Ecology of the Common Dolphin (*Delphinus delphis*) in Western Iberian Waters: Has the Decline in Sardine (*Sardina pilchardus*) Affected Dolphin Diet?” Marine Biology 165: 44. 10.1007/s00227-018-3285-3.

[ece371815-bib-0046] Marchand, M. L. , T. Hattab , N. Niquil , C. Albouy , F. L. Loc'h , and F. B. R. Lasram . 2020. “Climate Change in the Bay of Biscay: Changes in Spatial Biodiversity Patterns Could Be Driven by the Arrivals of Southern Species.” Marine Ecology Progress Series 647: 17–31. 10.3354/meps13401.

[ece371815-bib-0047] Meheust, E. , C. Dars , W. Dabin , et al. 2021. Les échouages de mammifères marins sur le littoral français en 2020. (Rapport scientifique de l'Observatoire Pelagis). La Rochelle Université et CNRS.

[ece371815-bib-0048] Meynier, L. , C. Pusineri , J. Spitz , M. Santos , G. Pierce , and V. Ridoux . 2008. “Intraspecific Dietary Variation in the Short‐Beaked Common Dolphin *Delphinus delphis* in the Bay of Biscay: Importance of Fat Fish.” Marine Ecology Progress Series 354: 277–287. 10.3354/meps07246.

[ece371815-bib-0049] Monteiro, S. , M. Ferreira , J. V. Vingada , A. López , A. Brownlow , and P. Méndez‐Fernandez . 2015. “Application of Stable Isotopes to Assess the Feeding Ecology of Long‐Finned Pilot Whale ( *Globicephala melas* ) in the Northeast Atlantic Ocean.” Journal of Experimental Marine Biology and Ecology 465: 56–63. 10.1016/j.jembe.2015.01.007.

[ece371815-bib-0050] Morissette, L. , V. Christensen , and D. Pauly . 2012. “Marine Mammal Impacts in Exploited Ecosystems: Would Large Scale Culling Benefit Fisheries?” PLoS One 7: e43966. 10.1371/journal.pone.0043966. 22970153 PMC3435392

[ece371815-bib-0051] Morizur, Y. , S. D. Berrow , N. J. C. Tregenza , A. S. Couperus , and S. Pouvreau . 1999. “Incidental Catches of Marine‐Mammals in Pelagic Trawl Sheries of the Northeast Atlantic.” Fisheries Research 41: 297–307.

[ece371815-bib-0052] Newsome, S. D. , M. T. Clementz , and P. L. Koch . 2010. “Using Stable Isotope Biogeochemistry to Study Marine Mammal Ecology.” Marine Mammal Science 26: 509–572. 10.1111/j.1748-7692.2009.00354.x.

[ece371815-bib-0053] Northridge, S. P. 1991. An Updated World Review of Interactions Between Marine Mammals and Fisheries. FAO Fisheries Technical Paper. Food & Agriculture Org.

[ece371815-bib-0054] Ogle, D. H. , J. C. Doll , A. P. Wheeler , and A. Dinno . 2025. FSA: Simple Fisheries Stock Assessment Methods.

[ece371815-bib-0055] Oksanen, J. , G. L. Simpson , F. G. Blanchet , et al. 2024. “vegan: Community Ecology Package.” 10.32614/CRAN.package.vegan.

[ece371815-bib-0056] Paillé, J. , C. Vignard , M. Authier , et al. 2024. “Identification of Static Netters Fishing Trajectories With High Resolution Data and Their Evolution in the Bay of Biscay Since 2015: Potential Implications for Short‐Beaked Common Dolphin Bycatch.” Fisheries Research 278: 107119. 10.1016/j.fishres.2024.107119.

[ece371815-bib-0057] Peet, R. K. 1975. “Relative Diversity Indices.” Ecology 56: 496–498. 10.2307/1934984.

[ece371815-bib-0058] Peltier, H. , M. Authier , F. Caurant , et al. 2021. “In the Wrong Place at the Wrong Time: Identifying Spatiotemporal Co‐Occurrence of Bycaught Common Dolphins and Fisheries in the Bay of Biscay (NE Atlantic) From 2010 to 2019.” Frontiers in Marine Science 8: 7342. 10.3389/fmars.2021.617342.

[ece371815-bib-0059] Peltier, H. , M. Authier , R. Deaville , et al. 2016. “Small Cetacean Bycatch as Estimated From Stranding Schemes: The Common Dolphin Case in the Northeast Atlantic.” Environmental Science & Policy 63: 7–18. 10.1016/j.envsci.2016.05.004.

[ece371815-bib-0060] Perrin, W. F. 2018. “Common Dolphin: *Delphinus delphis* .” In Encyclopedia of Marine Mammals, edited by B. Würsig , J. G. M. Thewissen , and K. M. Kovacs , Third ed., 205–209. Academic Press. 10.1016/B978-0-12-804327-1.00095-9.

[ece371815-bib-0062] R Core Team . 2023. R: A Language and Environment for Statistical Computing. R Foundation for Statistical Computing.

[ece371815-bib-0063] Read, A. J. 2008. “The Looming Crisis: Interactions Between Marine Mammals and Fisheries.” Journal of Mammalogy 89: 541–548. 10.1644/07-MAMM-S-315R1.1.

[ece371815-bib-0064] Santos, M. , I. German , D. Correia , et al. 2013. “Long‐Term Variation in Common Dolphin Diet in Relation to Prey Abundance.” Marine Ecology Progress Series 481: 249–268. 10.3354/meps10233.

[ece371815-bib-0065] Schoeman, R. P. , C. Patterson‐Abrolat , and S. Plön . 2020. “A Global Review of Vessel Collisions With Marine Animals.” Frontiers in Marine Science 7: 292. 10.3389/fmars.2020.00292.

[ece371815-bib-0066] Somerfield, P. J. , K. R. Clarke , and R. N. Gorley . 2021. “Analysis of Similarities (ANOSIM) for 2‐Way Layouts Using a Generalised ANOSIM Statistic, With Comparative Notes on Permutational Multivariate Analysis of Variance (PERMANOVA).” Austral Ecology 46: 911–926. 10.1111/aec.13059.

[ece371815-bib-0067] Soykan, C. , J. Moore , R. Zydelis , L. Crowder , C. Safina , and R. Lewison . 2008. “Why Study Bycatch? An Introduction to the Theme Section on Fisheries Bycatch.” Endangered Species Research 5: 91–102. 10.3354/esr00175.

[ece371815-bib-0068] Spitz, J. , T. Chouvelon , M. Cardinaud , C. Kostecki , and P. Lorance . 2013. “Prey Preferences of Adult Sea Bass *Dicentrarchus labrax* in the Northeastern Atlantic: Implications for Bycatch of Common Dolphin *Delphinus delphis* .” ICES Journal of Marine Science 70: 452–461. 10.1093/icesjms/fss200.

[ece371815-bib-0069] Spitz, J. , E. Mourocq , J.‐P. Leauté , J.‐C. Quéro , and V. Ridoux . 2010. “Prey Selection by the Common Dolphin: Fulfilling High Energy Requirements With High Quality Food.” Journal of Experimental Marine Biology and Ecology 390: 73–77. 10.1016/j.jembe.2010.05.010.

[ece371815-bib-0070] Spitz, J. , V. Ridoux , and A. Brind'Amour . 2014. “Let's Go Beyond Taxonomy in Diet Description: Testing a Trait‐Based Approach to Prey–Predator Relationships.” Journal of Animal Ecology 83: 1137–1148. 10.1111/1365-2656.12218. 24645977

[ece371815-bib-0505] Spitz, J. , V. Ridoux , A. W. Trites , S. Laran , and M. Authier . 2018. “Prey Consumption by Cetaceans Reveals the Importance of Energy‐Rich Food Webs in the Bay of Biscay.” Progress in Oceanography 166: 148–158.

[ece371815-bib-0071] Spitz, J. , A. W. Trites , V. Becquet , et al. 2012. “Cost of Living Dictates What Whales, Dolphins and Porpoises Eat: The Importance of Prey Quality on Predator Foraging Strategies.” PLoS One 7: e50096. 10.1371/journal.pone.0050096. 23185542 PMC3503768

[ece371815-bib-0072] Stelfox, M. , J. Hudgins , and M. Sweet . 2016. “A Review of Ghost Gear Entanglement Amongst Marine Mammals, Reptiles and Elasmobranchs.” Marine Pollution Bulletin 111: 6–17. 10.1016/j.marpolbul.2016.06.034. 27345709

[ece371815-bib-0073] Taboada, F. G. , G. Chust , M. Santos Mocoroa , et al. 2024. “Shrinking Body Size of European Anchovy in the Bay of Biscay.” Global Change Biology 30: e17047. 10.1111/gcb.17047. 38273534

[ece371815-bib-0074] Taylor, N. , M. Authier , R. Banga , M. Genu , K. MacLeod , and A. Gilles . 2022. “Marine Mammals by‐Catch.” In OSPAR 2023 : The 2023 Quality Status Report for the Northeast Atlantic. OSPAR Commission.

[ece371815-bib-0075] Trites, A. W. , and J. Spitz . 2018. “Diet.” In Encyclopedia of Marine Mammals, edited by B. Würsig , J. G. M. Thewissen , and K. M. Kovacs , Third ed., 255–259. Academic Press. 10.1016/B978-0-12-804327-1.00105-9.

[ece371815-bib-0076] Véron, M. , E. Duhamel , M. Bertignac , L. Pawlowski , and M. Huret . 2020. “Major Changes in Sardine Growth and Body Condition in the Bay of Biscay Between 2003 and 2016: Temporal Trends and Drivers.” Progress in Oceanography 182: 102274. 10.1016/j.pocean.2020.102274.

[ece371815-bib-0077] Wijnsma, G. , G. J. Pierce , and M. B. Santos . 1999. “Assessment of Errors in Cetacean Diet Analysis: *In Vitro* Digestion of Otoliths.” Journal of the Marine Biological Association of the United Kingdom 79: 573–575. 10.1017/S0025315498000733.

[ece371815-bib-0078] Wund, S. , E. Méheust , C. Dars , et al. 2023. “Strengthening the Health Surveillance of Marine Mammals in the Waters of Metropolitan France by Monitoring Strandings.” Frontiers in Marine Science 10: 1116819. 10.3389/fmars.2023.1116819.

[ece371815-bib-0079] Young, J. W. , B. P. V. Hunt , T. R. Cook , et al. 2015. “The Trophodynamics of Marine Top Predators: Current Knowledge, Recent Advances and Challenges.” Deep‐Sea Research Part II: Topical Studies in Oceanography 113: 170–187. 10.1016/j.dsr2.2014.05.015.

[ece371815-bib-0080] Žydelis, R. , C. Small , and G. French . 2013. “The Incidental Catch of Seabirds in Gillnet Fisheries: A Global Review.” Biological Conservation 162: 76–88. 10.1016/j.biocon.2013.04.002.

