# Supplementary material for: Long‐Term Changes in the Winter Diet of Common Dolphins Reflects Ecological Shifts and Bycatch Dynamics in the Bay of Biscay
Source: Ecol Evol. 2025 Jul 18;15(7):e71815. doi: 10.1002/ece3.71815 (PMC12271827; doi:10.1002/ece3.71815)
Supplement: Supplementary file 1 — Appendix S1. [file ECE3-15-e71815-s001.docx]

The winter diet of common dolphins over the last 20 years reflects prey and predator ecological changes in the Bay of Biscay, and highlights bycatch circumstances.

**Supplementary Material (Supplementary Table S1 and Figure S2)**

Johanna Faure^1^, Jasmin Niol^1^, Eléonore Meheust^1^, Jérôme Spitz^1,2^

^1^Observatoire Pelagis, UAR 3462 CNRS – La Rochelle Université, 5 allées de l’Océan, 17000 La Rochelle, France

^2^Centre d’Etudes Biologiques de Chizé (CEBC), UMR 7372 CNRS – La Rochelle Université, 79360, Villiers-en-Bois, France

Supplementary Table S1. Relationships and associated references used for the estimation of fish length (FL in cm) from otolith length (OL in mm) or from otolith weight (OW in g) and of fish mass (in g) from FL (in cm).

| **Prey species** |  | |  |  |  | | | | |  |
| --- | --- | --- | --- | --- | --- | --- | --- | --- | --- | --- |
|  | **Otolith – Fish length (cm)** | | | | | | | **Fish length (cm) – Fish body mass (g)** | | |
|  | Otolith length (OL) | | Otolith weight (OW) | | | | References | Fish length (FL) | References | |
| **FISH** | |  |  | | | |  |  |  | |
| **Caproidae** | |  |  | | | |  |  |  | |
| Capros aper | | 5,39xOL |  | | | <https://otoliths-northsea.linnaeus.naturalis.nl> | | 0,04717xFL^2,57^ | <https://fishbase.se> | |
| **Congridae** | |  |  | | | |  |  |  | |
| Conger conger | | (70,29xOL^1,13^)x0,1 |  | | | | Giménez et al., 2016 | 0,0004xFL^3,472^ | Mahé et al., 2016 | |
| **Argentinidae** | |  |  | | | |  |  |  | |
| Argentina spp. | | 3,87xOL |  | | | | <https://otoliths-northsea.linnaeus.naturalis.nl> (*Argentina sphyraena*) | 0,0021xFL^3,346^ | <https://fishbase.se>  (*Argentina sphyraena*) | |
| **Bathylagidae** | |  |  | | | |  |  |  | |
| Unidentified Bathylagidae | | 56,16975xOL - 39,7831 |  | | | | This study  (*Bathylachthys greyae*) | 0,0000105xFL^3,4460^ | This study  (*Bathylachthys greyae)* | |
| **Atherinidae** | |  |  | | | |  |  |  | |
| Atherina presbyter | | 3,11xOL |  | | | | <https://otoliths-northsea.linnaeus.naturalis.nl> | 0,0064xFL^3,05^ | <https://fishbase.se> | |
| **Belonidae** | |  |  | | | |  |  |  | |
| Belone belone | | 10,62xOL + 10,38 | 18,34xOW + 13,06 | | | | <https://otoliths-northsea.linnaeus.naturalis.nl> | 0,0011xFL^3,037^ | <https://fishbase.se> | |
| **Callionymidae** | |  |  | | | |  |  |  | |
| Callionymus spp. | | 8,41xOL -5,480 |  | | | | <https://otoliths-northsea.linnaeus.naturalis.nl> (*Callyonymus lyra*) | 0,00859xFL^2,927^ | <https://fishbase.se>  (*Callyonymus lyra*) | |
| **Carangidae** | |  |  | | | |  |  |  | |
| Trachurus spp. | | (15,76xOL + 1,39)x0,1 | (30,96xOW + 1,67)x0,1 | | | | Giménez et al., 2016  (*Trachurus trachurus*) | 0,0162xFL^2,83^ | Mahé et al., 2016 | |
| **Alosidae** | |  |  | | | |  |  |  | |
| Sardina pilchardus | | 8,13xOL - 6,09 | 16,88xOW - 5,96 | | | | <https://otoliths-northsea.linnaeus.naturalis.nl> | 0,00808xFL^3,009493^ | Santos et al., 2007 | |
| **Clupleidae** | |  |  | | | |  |  |  | |
| Clupea harengus | |  | 15,51xOW -6,360 | | | | <https://otoliths-northsea.linnaeus.naturalis.nl> | 0,0056xFL^3,037^ | Mahé et al., 2016 | |
| Sprattus sprattus | | 6,87xOL | 11,92xOW -1,410 | | | | <https://otoliths-northsea.linnaeus.naturalis.nl> | 0,0063xFL^3,032^ | Mahé et al., 2016 | |
| **Engraulidae** | |  |  | | | |  |  |  | |
| Engraulis encrasicolus | | (31,61xOL^1,25^)x0,1 | (86,34xOW^1,2^)x0,1 | | | | Giménez et al., 2016 | 0,00654xFL^2,981^ | <https://fishbase.se> | |
| Unidentified Clupeiformes | | 8,13xOL - 6,09 | 16,88xOW - 5,96 | | | | <https://otoliths-northsea.linnaeus.naturalis.nl> (*Sardina pilchardus*) | 0,00808xFL^3,009493^ | Santos et al., 2007 | |
| **Labridae** | |  |  | | | |  |  |  | |
| Unidentified Labridae | | (67,97xOL - 31,24)x0,1 | (86,34xOW^1,2^)x0,1 | | | | Härkönen et al., 1986  (*Labrus bergylta*) | 0,695xFL^4,205^ | Härkönen et al., 1986  (*Labrus bergylta*) | |
| **Sparidae** | |  |  | | | |  |  |  | |
| Boops boops | | (48,77xOL^0,8^)x0,1 |  | | | | Giménez et al., 2016 | 0,0083xFL^3,037^ | Kara & Bayhan, 2008 | |
| Sparidae spp. | | (20,21xOL^1,16^)x0,1 |  | | | | Giménez et al., 2016  (Spondyliosoma canthrus) | 0,0102xFL^3,152^ | Mahé et al., 2016 | |
| **Gadidae** | |  |  | | | |  |  |  | |
| Gadiculus argenteus | | (19,47xOL^0,89^)x0,1 |  | | | | Giménez et al., 2016 | 0,0021289xFL^10,0^ | Härkönen et al., 1986 | |
| Merlangius merlangus | | 1,73xOL + 0,81 | 6,74xOW - 2,97 | | | | <https://otoliths-northsea.linnaeus.naturalis.nl> | 0,0061xFL^3,095^ | Mahé et al., 2016 | |
| Micromesistius poutassou | | 2,66xOL - 5,65 | 9,11xOW - 9,65 | | | | <https://otoliths-northsea.linnaeus.naturalis.nl> | 0,0071xFL^2,988^ | Mahé et al., 2016 | |
| Trisopterus spp. | | 2,610xOL - 3,84 |  | | | | <https://otoliths-northsea.linnaeus.naturalis.nl>  (*Trisopterus minutus)* | 0,018xFL^2,81^ | Mahé et al., 2016  (*Trisopterus minutus)* | |
| Unidentified Gadidae | | 2,66xOL - 5,65 | 9,11xOW - 9,65 | | | | <https://otoliths-northsea.linnaeus.naturalis.nl>  (*Micromesistius poutassou*) | 0,0071xFL^2,988^ | Mahé et al., 2016  (*Micromesistius poutassou*) | |
| **Lotidae** | |  |  | | | |  |  |  | |
| Molva molva | | (51,94xOL^1,03^)x0,1 | (99,39xOW^1,03^)x0,1 | | | | Giménez et al., 2016  (*Molva* spp.) | 0,0031xFL^3,14^ | <https://fishbase.se>  (*Molva* spp.) | |
| **Gaidropsaridae** (Rockling) | |  |  | | | |  |  |  | |
| Unidentified Gaidropsaridae | | (32,96xOL^1,28)x0,1 |  | | | | Giménez et al., 2016  (*Gaidropsarus vulgaris*) | 0,01199xFL^2,547^ | <https://fishbase.se>  (*Gaidropsarus vulgaris*) | |
| **Merlucciidae** | |  |  | | | |  |  |  | |
| Merluccius merluccius | | 2,66xOL - 4,35 |  | | | | <https://otoliths-northsea.linnaeus.naturalis.nl> | 0,008xFL^2,964^ | Mahé et al., 2016 | |
| **Gobiidae** | |  |  | | | |  |  |  | |
| Aphia minuta | | 4,0xOL + 4,0 |  | | | | <https://otoliths-northsea.linnaeus.naturalis.nl> | 0,0039xFL^3,31 | https://fishbase.se | |
| Gobius spp. | | (29,09xOL^0,94)x0,1 |  | | | | Giménez et al., 2016  (*Gobius niger*) | 0,0082xFL^3,19 | <https://fishbase.se>  (*Gobius niger*) | |
| Lesueurigobius friesii | | 2,1817xOL - 0,1263 |  | | | | Daban et al., 2020 | 0,0087xFL^2,9630 | https://fishbase.se | |
| Pomatoschistus spp. | | 3,92xOL - 0,43 |  | | | | <https://otoliths-northsea.linnaeus.naturalis.nl> (*Pomatoschistus minutus*) | 0,00622xFL^3,173 | <https://fishbase.se>  (*Pomatoschistus minutus*) | |
| Unidentified Gobiidae | | 3,92xOL - 0,43 |  | | | | <https://otoliths-northsea.linnaeus.naturalis.nl>  (*Pomatoschistus minutus*) | 0,00622xFL^3,173 | <https://fishbase.se>  (*Pomatoschistus minutus*) | |
| **Mugilidae** | |  |  | | | |  |  |  | |
| Unidentified Mugilidae | | 4,30xOL - 4,85 |  | | | | <https://otoliths-northsea.linnaeus.naturalis.nl>  (*Liza ramada*) | 0,008xFL^3,15 | <https://fishbase.se>  (*Liza ramada*) | |
| **Mullidae** | |  |  | | | |  |  |  | |
| Mullus surmuletus | | (26,37xOL^1,57)x0,1 |  | | | | Giménez et al., 2016 | 0,0069xFL^3,219 | <https://fishbase.se> | |
| **Myctophidae** | |  |  | | | |  |  |  | |
| Benthosema glaciale | | (37,97xOL^1,3)x0,1 |  | | | | Giménez et al., 2016 | 0,0106xFL^2,916 | <https://fishbase.se> | |
| Ceratoscopelus maderensis | | 12,417xOL + 15,858 |  | | | | Battaglia et al., 2010 | 0,0000074xFL^3,144 | Battaglia et al., 2010 | |
| Myctophum punctatum | | (20,85xOL^1,24)x0,1 |  | | | | Giménez et al., 2016 | 0,0055xFL^3,22 | <https://fishbase.se> | |
| Notoscopelus kroeyeri | | 21,424xOL - 7,2438 |  | | | | This study | 0,0399xFL^3,5475 | This study | |
| **Ammodytidae** | |  |  | | | |  |  |  | |
| Unidentified Ammodytidae | | 6,8xOL - 2,56 |  | | | | <https://otoliths-northsea.linnaeus.naturalis.nl> (*Hyperoplus lanceolatus*) | 0,0101xFL^2,671 | Mahé et al., 2016  (*Hyperoplus immaculatus*) | |
| **Flatfish** | |  |  | | | |  |  |  | |
| **Bothidae** | |  |  | | | |  |  |  | |
| Arnoglossus spp. | | 5,64xOL - 0,770 |  | | | | <https://otoliths-northsea.linnaeus.naturalis.nl>  (*Arnoglossus laterna*) | 0,0063xFL^3,084 | <https://fishbase.se>  (*Arnoglossus laterna*) | |
| Unidentified Bothidae | | 5,5xOL |  | | | | <https://otoliths-northsea.linnaeus.naturalis.nl>  (*Phrynorhombus norvegicus*) | 0,0103xFL^3,054 | <https://fishbase.se>  (*Phrynorhombus norvegicus*) | |
| **Scophthalmidae** | |  |  | | | |  |  |  | |
| Unidentified Scophthalmidae | | 6,49xOL - 2,93 |  | | | | <https://otoliths-northsea.linnaeus.naturalis.nl>  (*Scophthalmus rhombus*) | 0,0088xFL^3,124 | Mahé et al., 2016  (*Scophthalmus rhombus*) | |
| **Soleidae** | |  |  | | | |  |  |  | |
| Dicologlossa cuneata | | 64,6330xOL - 37,9820 |  | | | | This study | 0,9185xFL^3,3248 | This study | |
| Microchirus spp. /Buglossidium spp. | | (59,16xOL^1,09)x0,1 |  | | | | Giménez et al., 2016  (*Pegusa lascaris*) | 0,000782xFL^3,128 | <https://fishbase.se>  (*Buglossidium luteum*) | |
| Pegusa lascaris | | (59,16xOL^1,09)x0,1 |  | | | | Giménez et al., 2016 | 0,000782xFL^3,128 | <https://fishbase.se>  (*Buglossidium luteum*) | |
| Solea spp. | | 8,18xOL - 2,650 |  | | | | <https://otoliths-northsea.linnaeus.naturalis.nl>  (*Solea solea*) | 0,0039xFL^3,251 | Mahé et al., 2016  (*Solea solea*) | |
| **Scombridae** | |  |  | | | |  |  |  | |
| Scomber spp. | | (87,59xOL - 20,41)x0,1 | (153,49xOW^0,93)x0,1 | | | | Härkönen et al., 1986 / Giménez et al., 2016  (*Scomber scombrus*) | 0,00422xFL^3,26 | <https://fishbase.se>  (*Scomber scombrus*) | |
| **Sternoptychidae** | |  |  | | | |  |  |  | |
| Maurolicus muelleri | | 2,739xOL - 22,842 |  | | | | Battaglia et al., 2010 | 0,000016xFL^2,946 | Battaglia et al., 2010 | |

^
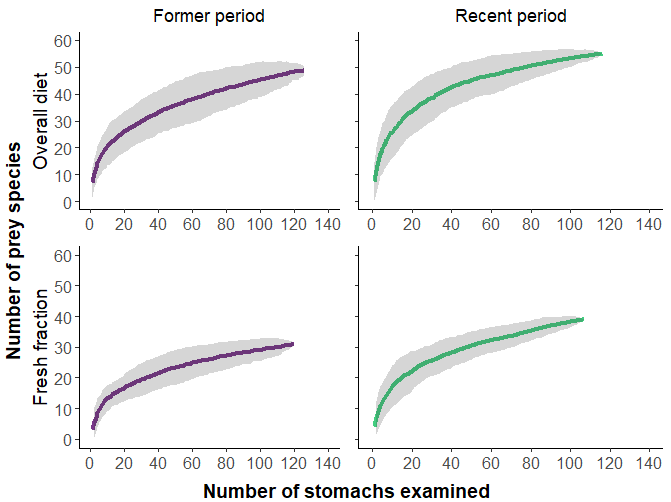
^

Supplementary Figure S2. Cumulative prey curves (solid line) and confidence interval of 95% upper and lower (grey area) where b ≤ 0.05 through the last five subsamples.

**References**

Battaglia, P., Malara, D., Romeo, T., Andaloro, F., 2010. Relationships between otolith size and fish size in some mesopelagic and bathypelagic species from the Mediterranean Sea (Strait of Messina, Italy). Sci. Mar. 74, 605–612. https://doi.org/10.3989/scimar.2010.74n3605

Daban, İ.B., Arslan İhsanoglu, M., İsmen, A., 2020. Relationships between body size - otolith size for seven demersal fish species from the Marmara Sea, Turkey. Ege J. Fish. Aquat. Sci. 37, 267–274. https://doi.org/10.12714/egejfas.37.3.09

Giménez, J., Manjabacas, A., Tuset, V.M., Lombarte, A., 2016. Relationships between otolith and fish size from Mediterranean and north-eastern Atlantic species to be used in predator–prey studies. J. Fish Biol. 89, 2195–2202. https://doi.org/10.1111/jfb.13115

Härkönen, T., 1986. Guide to the otoliths of the bony fishes of the Northeast Atlantic, Danbiu ApS. ed.

Kara, A., Bayhan, B., 2008. Length-weight and length-length relationships of the bogue Boops boops (Linneaus, 1758) in Izmir Bay (Aegean Sea of Turkey).

Mahé, K., Bellamy, E., Delpech, J.P., Lazard, C., Salaun, M., Vérin, Y., Coppin, F., Travers-Trolet, M., 2018. Evidence of a relationship between weight and total length of marine fish in the North-eastern Atlantic Ocean: physiological, spatial and temporal variations. J. Mar. Biol. Assoc. U. K. 98, 617–625. https://doi.org/10.1017/S0025315416001752

Santos, M.B., Fernández, R., López, A., Martínez, J.A., Pierce, G.J., 2007. Variability in the diet of bottlenose dolphin, *Tursiops truncatus*, in Galician waters, north-western Spain, 1990–2005. J. Mar. Biol. Assoc. U. K. 87, 231–241. https://doi.org/10.1017/S0025315407055233
